# *GPR116* receptor regulates the antitumor function of NK cells via Gαq/HIF1α/NF-κB signaling pathway as a potential immune checkpoint

**DOI:** 10.1186/s13578-023-01005-7

**Published:** 2023-03-09

**Authors:** Dandan Guo, Chenxu Jin, Yaoxin Gao, Haizhen Lin, Li Zhang, Ying Zhou, Jie Yao, Yixin Duan, Yaojun Ren, Xinhui Hui, Yujia Ge, Renzheng Yang, Wenzheng Jiang

**Affiliations:** Shanghai Key Laboratory of Regulatory Biology, School of Life Sciences, East China Normal University, 500 Dongchuan Rood, Shanghai, 200241 China

**Keywords:** GPR116, NK, HIF1α, NK-κB, Immunotherapy

## Abstract

**Background:**

NK cell is one of innate immune cells and can protect the body from cancer-initiating cells. It has been reported that *GPR116* receptor is involved in inflammation and tumors. However, the effect of *GPR116* receptor on the NK cells remains largely unclear.

**Results:**

We discovered that *GPR116*^*−/−*^ mice could efficiently eliminate pancreatic cancer through enhancing the proportion and function of NK cells in tumor. Moreover, the expression of *GPR116* receptor was decreased upon the activation of the NK cells. Besides, *GPR116*^*−/−*^ NK cells showed higher cytotoxicity and antitumor activity in vitro and in vivo by producing more GzmB and IFNγ than wild-type (WT) NK cells. Mechanistically, *GPR116* receptor regulated the function of NK cells via Gαq/HIF1α/NF-κB signaling pathway. Furthermore, downregulation of *GPR116* receptor promoted the antitumor activity of NKG2D-CAR-NK92 cells against pancreatic cancer both in vitro and in vivo.

**Conclusions:**

Our data indicated that GPR116 receptor had a negatively effect on NK cell function and downregulation of GPR116 receptor in NKG2D-CAR-NK92 cells could enhance the antitumor activity, which provides a new idea to enhance the antitumor efficiency of CAR NK cell therapy.

**Supplementary Information:**

The online version contains supplementary material available at 10.1186/s13578-023-01005-7.

## Background

G protein-coupled receptors (GPCRs) are the largest class of cell surface receptors in the genome, which recognize a wide variety of extracellular molecules, such as neurotransmitters, hormones, light, and odors [1]. GPCRs are involved in a number of physiological and pathological processes. According to statistics, GPCRs are the most successful family of targets of FDA-approved drugs. Therefore, exploring the ability to quantify GPCR expression and ligand binding characteristics in different cell types and tissues is very important for drug discovery [2].

Adhesive GPCRs (aGPCRs) belong to the second largest family of GPCRs. It is named for its longer adhesion terminal and includes 33 members in humans and 30 members in mice, including *GPR116* (ADGRF5) [3, 4]. *GPR116* receptor are widely expressed in a variety of tissues and organs, such as lung, heart, liver, kidney and spleen [5]. According to the expression distribution and functional characteristics of *GPR116* receptor, studies have shown that *GPR116* receptor is involved in the function of vascular skin cells [6], regulates pulmonary homeostasis [7, 8], and plays an important role in the development of tumors [9, 10]. There has been reported that the structure of the N-terminal of *GPR116* receptor is very similar to that of the LN-7TM receptor, belonging to a new GPCR of the LN-7TM receptor subgroup [11] and the N-terminal of *GPR116* receptor contains two C-like immunoglobulin-like functional domains. Immunoglobulin plays an important role in the immune system and intercellular interactions [12, 13], suggesting that *GPR116* receptor may have the function in regulating the immune system. At present, there are few reports on the regulatory mechanism of *GPR116* receptor in the immune system, but *GPR116* receptor may play a significant role in regulating the immune response, which has been repeatedly mentioned in the relevant literatures [14, 15].

NK cell is a special killer cell because of its natural killing characteristics, which can eliminate abnormal cells damaged by malignant transformation or virus infection, and their activity is regulated by an array of activating and inhibitory receptors. At the same time, monoclonal antibody (mAb) therapy has been used to activate NK cell-mediated antibody-dependent cytotoxicity (ADCC) in the treatment of solid cancer [16]. It has been reported that NK cells can infiltrate into solid tumors, metastatic tumors and tumor infiltrating lymph nodes [17]. It was found that the deletion of inhibitory receptor NKR-P1B promoted the expression of GzmB in NK cells and enhanced NK cell-mediated antitumor effects [18]. In recent years, the treatment of chimeric antigen receptor NK (CAR-NK) cells and CAR-T cells are also the hot spot of antitumor research [19, 20]. CAR-NK and CAR-T cells are mainly based on their precise and efficient antitumor effects of extracellular targeting and intracellular costimulatory molecules. To date, two CD19-CAR-T cell therapies have been approved for the treatment of acute lymphoblastic leukemia (ALL) and diffuse large B-cell lymphoma (DLBCL) [21]. However, CAR-T cell therapy still faces several problems. Cytokine release syndrome (CRS) and neurotoxicity (NT) are serious side effects in CAR-T cell therapy. All these factors may limit the further clinical application of CAR-T cell therapy [22]. Therefore, we investigated whether *GPR116* receptor affects the function of NK cells and can be applied to the tumor treatment of CAR-NK cells.

Here our data demonstrated that *GPR116* receptor was negatively correlated with the activation of NK cells. Downregulation of *GPR116* receptor promoted the anti-pancreatic cancer function of NK cells through Gαq/HIF1α/NF-κB signaling pathway. Furthermore, we downregulated *GPR116* receptor in NKG2D-CAR-NK92 cells, and the results showed that *GPR116* receptor depletion could effectively enhance the antitumor function of NKG2D-CAR-NK92 cells. In a word, our study elucidated the function of *GPR116* receptor in NK cells, and provided a new idea to enhance antitumor efficiency of CAR-NK cell therapy.

## Results

### Deletion of GPR116 receptor inhibits pancreatic cancer growth by promoting NK cell infiltration and function

**Fig. 1:**
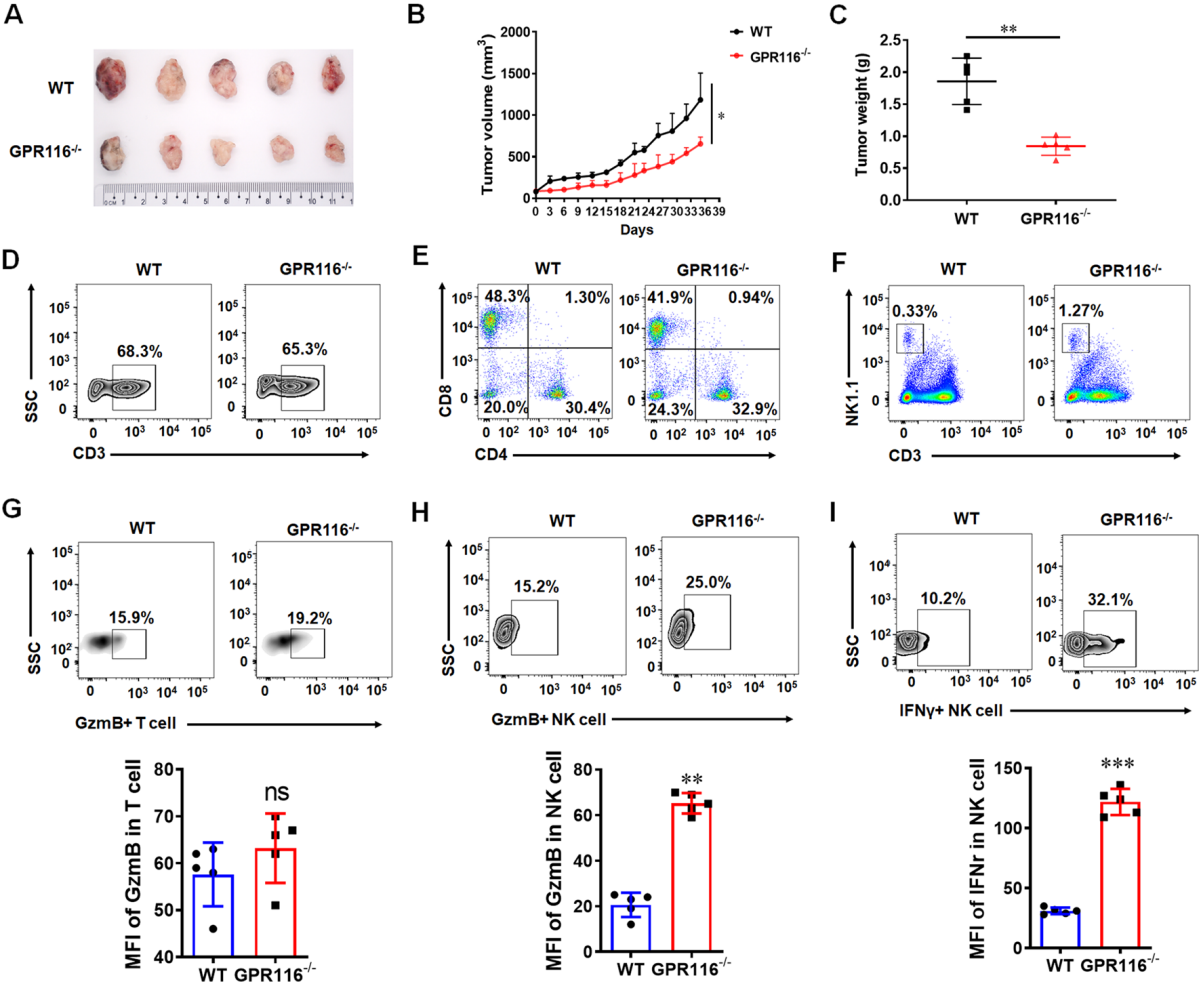
Knockout of *GPR116* inhibits pancreatic cancer growth in mice by affecting the infiltration and function of NK cells. **A** The photo of PAAD tumor sizes (n = 5). **B** The PAAD tumor growth curves and the end-point tumor sizes were represented (n = 5). **C** The weight of PAAD tumor (n = 5). **D** The infiltration of CD3^+^ T cells in PAAD tumor. **E** The proportion of CD4^+^ and CD8^+^ T cells in PAAD tumor. **F** The infiltration of CD3^−^ NK1.1^+^ cells in PAAD tumor. **G** The expression of GzmB in tumor-infiltrating CD3^+^ T cells. **H** The expression of GzmB in tumor-infiltrating CD3^−^ NK1.1^+^ cells. **I** The expression of IFNγ in tumor-infiltrating CD3^−^ NK1.1^+^ cells. All data are from at least three independent experiments. Data are represented as the mean ± standard error of the mean (SEM). *ns* no significance, *P < 0.05, **P < 0.01, ***P < 0.001 by an unpaired Student’s t-test

Our results showed that the proportion of NK cells increased significantly after *GPR116* receptor deletion including bone marrow, spleen, lymph nodes and lungs (Additional file 1: Fig. S1A–E). Then, the wild-type (WT) and *GPR116* receptor-deficient mice were used for subcutaneous tumor model of pancreatic cancer. The proportion of tumor-infiltrating lymphocytes and the expression level of cytokines in lymphocytes were analyzed. The results showed that the deletion of *GPR116* receptor inhibited the growth of pancreatic cancer (Fig. 1A–C). The proportion of CD3^+^ T, CD4^+^ T, CD8^+^ T and NK cells, and the expression level of granzyme B (GzmB) and interferon γ (IFNγ) were analyzed by flow cytometry in CD8^+^ T and NK cells. It was found that the proportion of CD3^+^ T, CD4^+^ T and CD8^+^ T in pancreatic cancer did not change significantly (Fig. 1D, E), and there was no significant difference in the expression level of GzmB in CD8^+^ T after the deletion of *GPR116* receptor (Fig. 1G). However, after the deletion of *GPR116* receptor, the proportion of NK cells in pancreatic cancer increased significantly (Fig. 1F), and the expression level of GzmB and IFNγ in NK cells were also increased (Fig. 1H, I). These results suggested that *GPR116* receptor may regulate NK cell function.

### Downregulation of GPR116 receptor promotes the killing function of NK92 cells

**Fig. 2:**
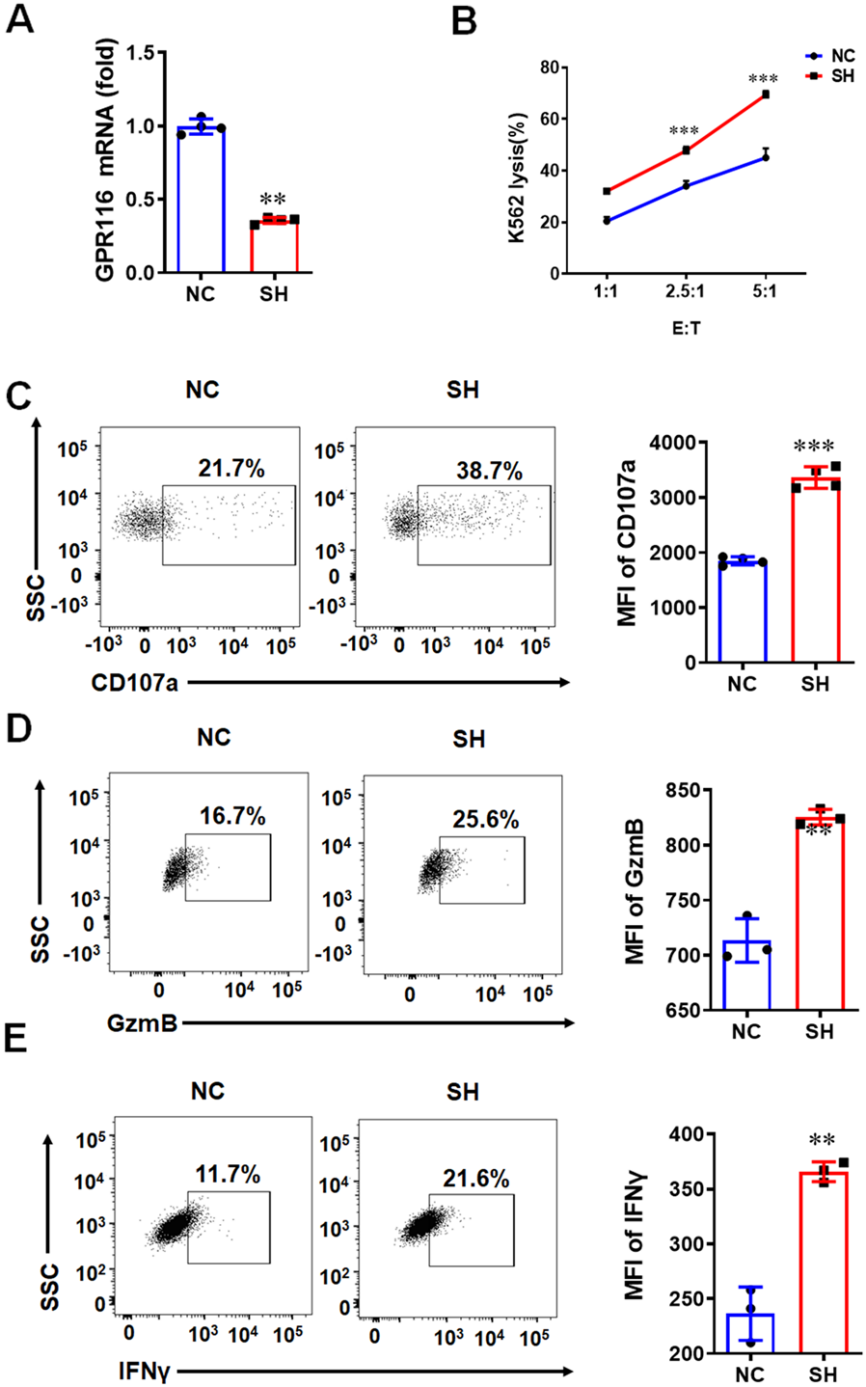
Downregulation of *GPR116* receptor enhances the function of NK92 cells. **A** qRT-PCR analysis of the expression of *GPR116* in NK92 cells after the lentivirus infection with shRNA. **B** The line plots displayed the cytotoxicity of NK92 cells (NC) and NK92 cells with *GPR116* knockdown (SH) against K562 cells at a different effector to target (E: T) ratios for 4 h. **C** Flow cytometry analysis of the expression of CD107a in NK92 cells after co-incubating with K562 cells at a 5:1 ratio for 4 h. **D** Flow cytometry analysis of the expression of GzmB in NK92 cells after co-incubating with K562 cells at a 5:1 ratio for 4 h. **E** Flow cytometry analysis of the expression of IFNγ in NK92 cells after co-incubating with K562 cells at a 5:1 ratio for 4 h. Data are represented as the mean ± standard error of the mean (SEM). **P < 0.01, ***P < 0.001 by an unpaired Student’s t-test

The activation of NK cells can be stimulated by interleukin and other external factors, and the activated NK cells show stronger killing ability [23]. Firstly, we detected the expression of *GPR116* receptor after NK cell activation. It was found that the mRNA levels of *GPR116* receptor were decreased after NK92 cell activation by hIL-15 (Additional file 1: Fig. S2A). At the same time, we isolated NK cells of WT mice, and stimulated them with mIL-15. After the activation of NK cells, the expression of *GPR116* receptor was decreased (Additional file 1: Fig. S2B), which was consistent with those of NK92 cells. Conversely, in order to understand whether the deletion of *GPR116* receptor will affect the activation of NK cells, we isolated the NK cells from WT and *GPR116*^−/−^ mice, and detected the mRNA levels of the activating receptor and inhibitory receptor of NK cells. The results showed that the mRNA levels of activating receptors NKG2D and NKp46 were increased, while the mRNA levels of inhibitory receptors NKG2A and KLRG1 were decreased after the deletion of *GPR116* receptor (Additional file 1: Fig. S2C–F). The above results showed that *GPR116* receptor was negatively correlated with the activation of NK cells. The activation of NK cells can improve the cytotoxicity of NK cells. To understand whether *GPR116* receptor affects the killing function of NK cells, we downregulated the expression of *GPR116* receptor in NK92 cells (Fig. 2A) and detected the apoptosis of K562 cells after co-culturing with NK92 cells. The results showed that *GPR116* receptor interference in NK92 cells could increase the antitumor activity against K562 cells compared to NK92 cells at 2.5:1 and 5:1 of effector and target cell ratio (Fig. 2B). After downregulating *GPR116* receptor, the expression of CD107a in NK92 cells increased significantly (Fig. 2C). Moreover, the expression of GzmB and IFNγ were also increased in NK92 cells after down regulating *GPR116* receptor (Fig. 2D, E). In addition, the similar results were verified in isolated mouse NK cells (Additional file 1: Fig. S3A–D). In a word, our data demonstrated that downregulation of *GPR116* receptor could enhance the antitumor function of NK cells.

### Downregulation of GPR116 receptor promotes the killing function of NK cells through Gαq/HIF1α/NF-κB signaling pathway

**Fig. 3:**
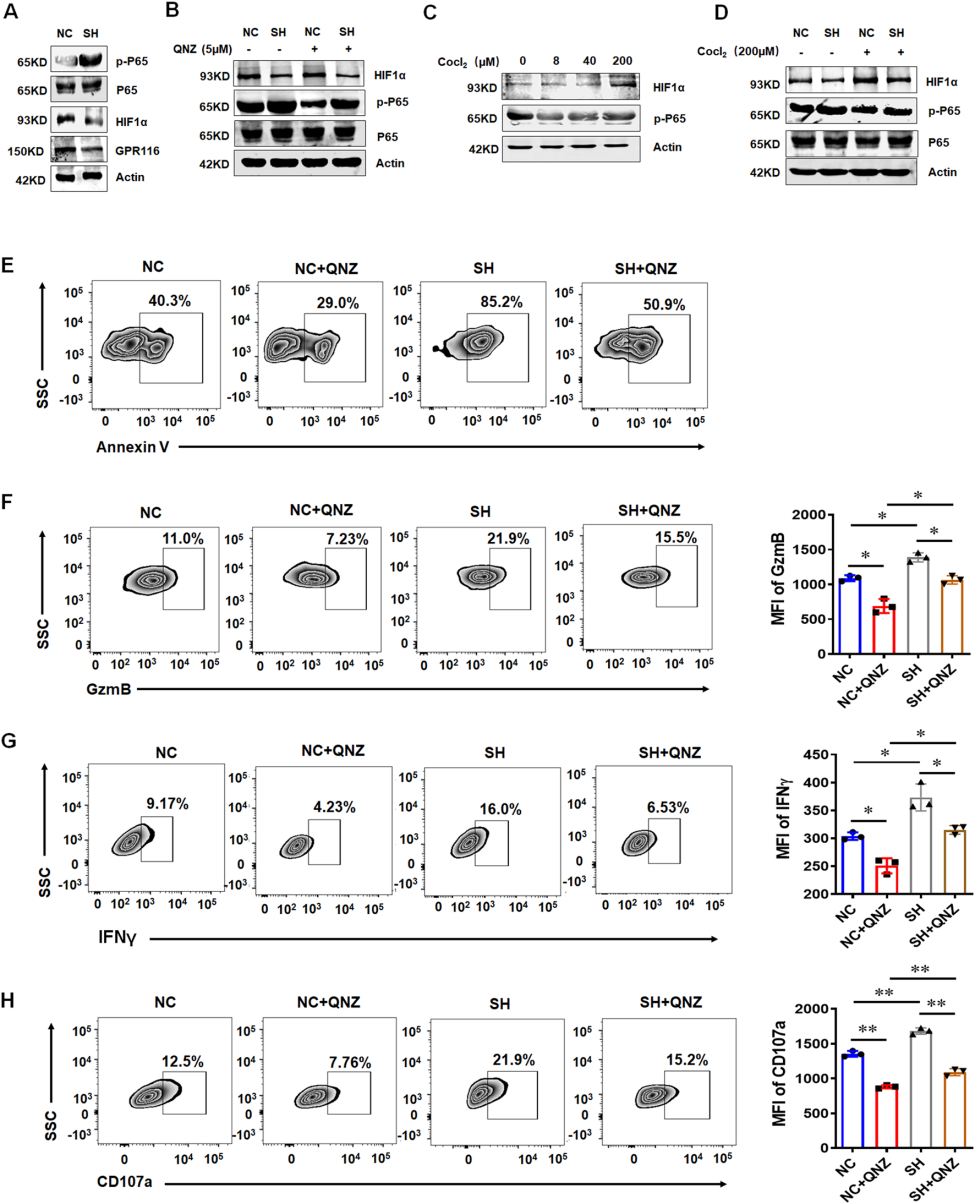
*GPR116* receptor regulates the function of NK92 cells via HIF1α/NF-κB signaling pathway. **A** Western blot analysis of p-P65 and HIF1α in NK92 cells (NC) and *GPR116*-knockdown NK92 cells (SH). **B** Western blot analysis of p-P65 and HIF1α in NK92 cells (NC) and *GPR116*-knockdown NK92 cells (SH) with or without QNZ. **C**, **D** Western blot analysis of p-P65 and HIF1α in NK92 cells after the treatment with CoCl_2_. **E** Flow cytometry analysis of the killing ability of NK92 cells (NC) and *GPR116*-knockdown NK92 cells (SH) with or without QNZ. Flow cytometry analysis of the expression of GzmB (**F**), IFNγ (**G**) and CD107a (**H**) in NK92 cells (NC) and *GPR116*-knockdown NK92 cells (SH) with or without QNZ. All data are from at least three independent experiments. Data are represented as the mean ± standard error of the mean (SEM). *P < 0.05, **P < 0.01 by an unpaired Student’s t-test

NF-κB signaling pathway can widely regulate numerous genes, including inflammatory factors, immune-related receptors and adhesion molecules [24]. It is reported that NF-κB signaling pathway plays an important role in the functional regulation of NK cells, and the upregulation of HIF1α in NK cells inhibited the activation of NF-κB signaling pathway to inhibit the function of NK cells in tumors [25]. Our results showed that the expression of *GPR116* receptor was positively correlated with the expression of HIF1α through TIMER 2.0 (Additional file 1: Fig. S4A). We first detected the expression of HIF1α in NK92 cells stimulated by hIL-15, the data showed that the expression of HIF1α was decreased in activated NK92 cells (Additional file 1: Fig. S4B). The similar results were found in the mouse NK cells after the treatment with mIL-15 (Additional file 1: Fig. S4C). To verify the relationship of *GPR116* receptor and HIF1α, we detected the expression of HIF1α in NK92 cells after downregulating the expression of *GPR116* receptor, the results showed that HIF1α expression was decreased after knockdown of *GPR116* receptor (Additional file 1: Fig. S4D). Furtherly, NF-κB luciferase reporter gene system was used to detect NF-κB signaling pathway in NK cells. It was found that downregulation of *GPR116* receptor activated NF-κB signaling pathway (Additional file 1: Fig. S4E). Next, we detected the protein level of HIF1α, total P65 and phosphorylated P65 in NK92 cells by western blot (WB). It was found that lower HIF1α and higher phosphorylation of P65 were observed after downregulation of *GPR116* receptor expression (Fig. 3A). Then we used NF-κB signaling pathway inhibitor QNZ to suppress the activity. The results showed that inhibition of p65 phosphorylation did not affect the expression of HIF1α (Fig. 3B). To mimic the tumor hypoxia microenvironment, NK92 cells were cultured in existence of CoCl_2_. The results showed that HIF1α expression was upregulated, and the induction of HIF1α expression could inhibit the phosphorylation of P65 (Fig. 3C, D). At the same time, we detected the killing function and the expression of CD107a, GzmB and IFNγ with or without QNZ in NK92 cells, the results showed that the NK92 killing function reduced (Fig. 3E) and the expression of CD107a, GzmB and IFNγ was downregulated when the NK-κB signaling pathway was inhibited (Fig. 3F–H). Tang X et al. showed that *GPR116* receptor affects the metastasis of breast cancer through Gαq/11 [6]. In current study, we also detected the effect of Gαq on the protein level of HIF1α and p65 phosphorylation. The data showed that knockdown of Gαq could reduce the expression of HIF1α and increase the phosphorylation of p65 (Additional file 1: Fig. S5A, B), the killing ability of NK92 and the expression of GzmB, CD107a and IFNγ also increased, the results were similar to knockdown of *GPR116* receptor (Additional file 1: Fig. S5C–E) [26]. These data indicated that *GPR116* receptor affected the function of NK cells through Gaq/HIF1α/NK-κB signaling pathway.

### GPR116 receptor-deficient NK cells inhibits the growth of pancreatic cancer

**Fig. 4:**
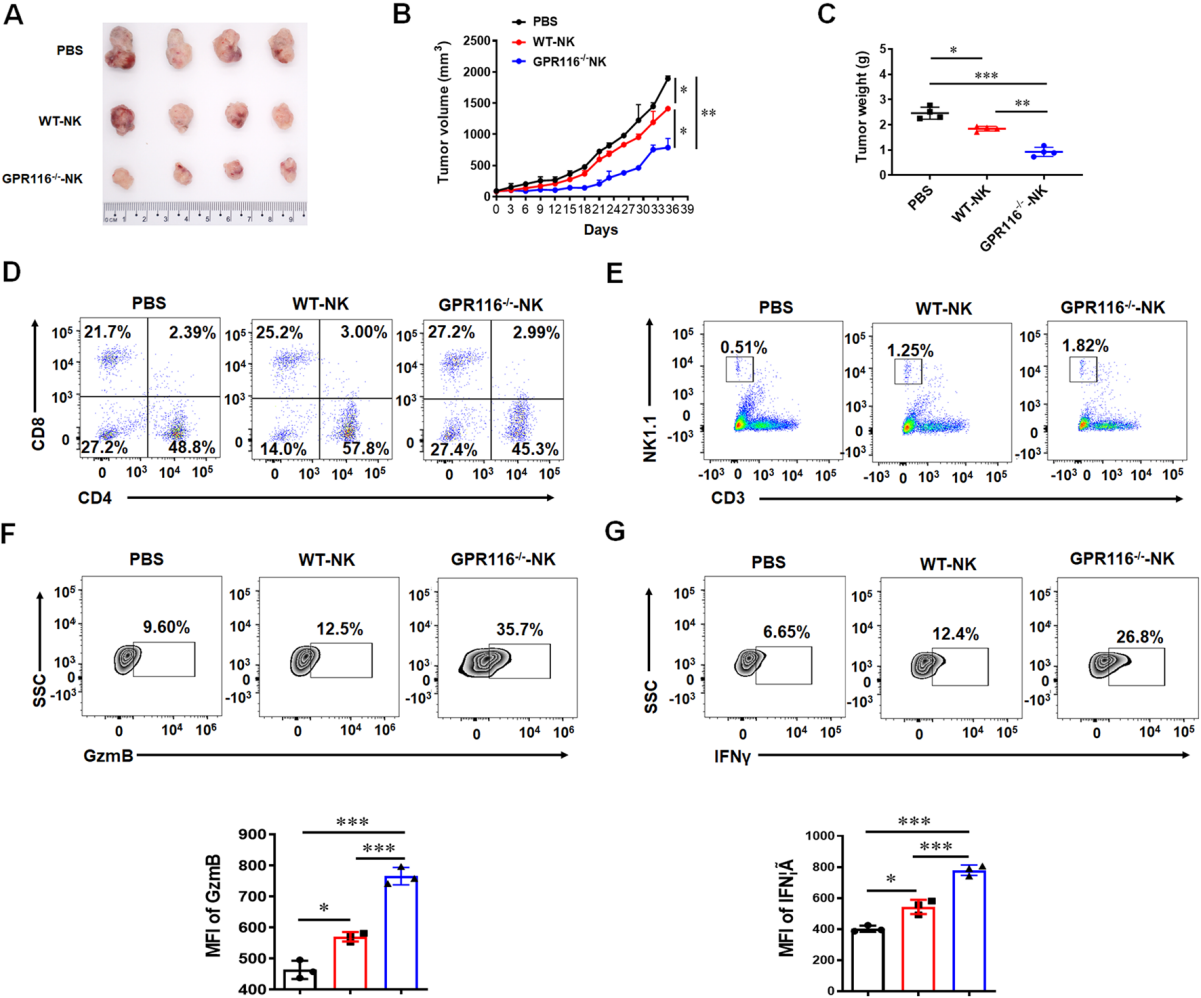
Deficiency of *GPR116* receptor enhances the antitumor effect of NK cells in vivo. Subcutaneous tumor models were established with PAN02 cells and these mice were injected with PBS, WT-NK and *GPR116*^−/−^-NK through tail vein, respectively. **A** The photo of PAAD tumor sizes (n = 4). **B** The PAAD tumor growth curves and the end-point tumor sizes were represented (n = 4). **C** The weight of PAAD tumor (n = 4). **D** The proportion of CD4^+^ and CD8^+^ T cells in PAAD tumor. **E** The infiltration of CD3^−^ NK1.1^+^ cells in PAAD tumor. **F** The expression of GzmB in tumor-infiltrating CD3^−^ NK1.1^+^ cells. **G** The expression of IFNγ in tumor-infiltrating CD3^−^ NK1.1^+^ cells. Data are represented as the mean ± standard error of the mean (SEM). *P < 0.05, **P < 0.01, ***P < 0.001 by an unpaired Student’s t-test

To explore the effect of *GPR116* receptor on antitumor, we constructed a subcutaneous model of pancreatic cancer in mice. After the tumor model mice were treated with PBS, WT-NK or *GPR116*^−/−^-NK cells, the tumor size and the immune cells in infiltrating tumors were analyzed. The statistical results demonstrated that *GPR116*^−/−^-NK cells could significantly suppress tumor growth (Fig. 4A–C). Flow analysis showed that there was no significant difference between CD8^+^ T and CD4^+^ T cells (Fig. 4D). The proportion of NK cells (Fig. 4E) and the expression of GzmB (Fig. 4F) and IFNγ (Fig. 4G) in *GPR116*^−/−^-NK group were significantly increased. These data indicated that *GPR116* receptor depletion promoted the anti-pancreatic cancer function of NK cells in vivo.

### Downregulation of GPR116 receptor enhances cytotoxicity of NKG2D-CAR-NK cells

**Fig. 5:**
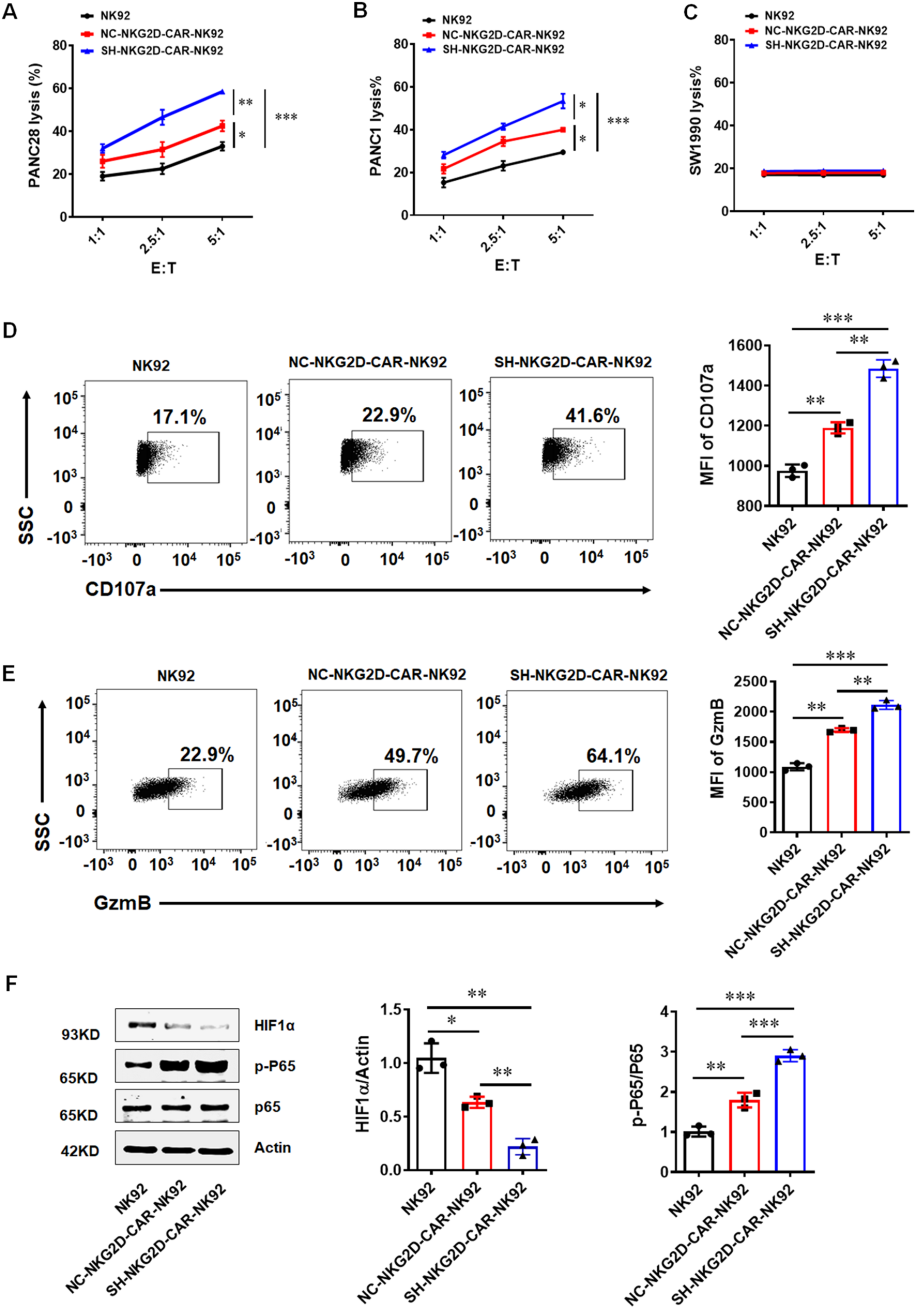
Downregulation of *GPR116* receptor promotes the antitumor activity of NKG2D-CAR-NK92 cells in vitro. **A**–**C** The line plots displayed the cytotoxicity of NK92 cells, NC-NKG2D-CAR-NK92 cells (normal control) and SH-NKG2D-CAR-NK92 cells (NKG2D-CAR-NK92 cells with *GPR116* knockdown) against PANC28 cells, PANC1 cells and SW1990 cells at a different effector to target (E: T) ratios for 4 h. **D** Flow cytometry analysis of the expression of CD107a in NK92 cells after co-incubating with PANC28 cells at a 5:1 ratio for 4 h. **E** Flow cytometry analysis of the expression of GzmB in NK92 cells after co-incubating with PANC28 cells at a 5:1 ratio for 4. **F** The expression of HIF1α and p-P65 in NKG2D-CAR-NK92 cells by WB. Data are represented as the mean ± standard error of the mean (SEM). *P < 0.05, **P < 0.01, ***P < 0.001 by an unpaired Student’s t-test

In order to better apply our research to clinical application, we constructed NKG2D-CAR with *GPR116* receptor knockdown (Additional file 1: Fig. S6A, B). Moreover, we detected the expression of NKG2D ligand MICA/B in pancreatic cancer cells to obtain the optimal target cells. Our data suggested that PANC28 cells and PANC1 cells showed higher expression of MICA/B, while the MICA/B expression was the lowest in SW1990 cells (Additional file 1: Fig. S7). NK92 cells, NC-NKG2D-CAR-NK92 cells and SH-NKG2D-CAR-NK92 cells were incubated with PANC28 cells, PANC1 cells and SW1990 cells respectively for 4 h at effector to target cell ratio of 1:1, 2.5:1 and 5:1. The data suggested that all types of NKG2D-CAR-NK92 cells exerted significantly cytotoxic activity against PANC28 and PANC1 but not SW1990 (Fig. 5A–C). As expected, downregulating of *GPR116* receptor in NKG2D-CAR-NK92 cells revealed higher cytotoxic activity against PANC28 and PANC1 compared to NC-NKG2D-CAR-NK92 cells. We next explored the expression of CD107a, an activation marker for NK-cell function [27]. A higher level of CD107a-positive cells was observed of NKG2D-CAR-NK cells in response to PANC28 and PANC1 cells but not SW1990 cells (Fig. 5D, Additional file 1: Fig. S8A, S9A). Furthermore, GzmB is also pivotal for cytolytic function of NK cells [28, 29]. The results demonstrated that NKG2D-CAR-NK92 cells produced more GzmB than non-transduced NK92 cells when co-cultured with PANC28 and PANC1 cells but not SW1990 cells (Fig. 5E, Additional file 1: Fig. S8B, S9B). Mechanically, *GPR116* receptor regulated the function of NKG2D-CAR-NK92 cells via HIF1α/NF-κB signaling pathway (Fig. 5F).

### Knockdown of GPR116 receptor improves antitumor activity of NKG2D-CAR-NK92 cells against xenograft pancreatic tumor model

**Fig. 6:**
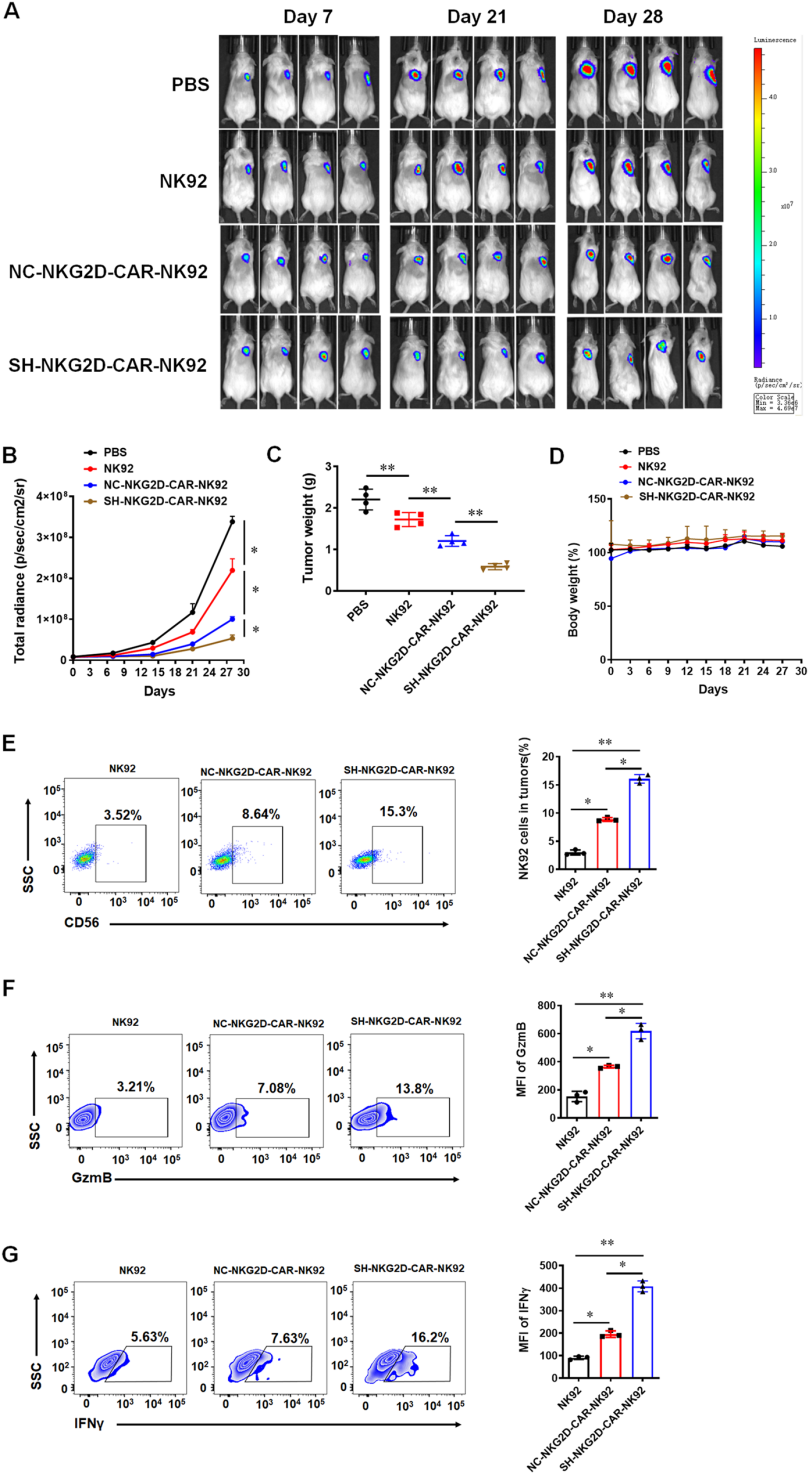
Downregulation of *GPR116* enhances antitumor activity of NKG2D-CAR-NK92 cells against xenografts formed by PANC28 in mice. **A**, **B** Tumor bioluminescence images of mice transplanted with PANC28/luc cells at the indicated time points. **C**, **D** Line plots displaying the tumor weight and the body weight of mice. **E** The proportion of NK92 cells in tumors was measured by flow cytometry. **F** The expression of GzmB in tumor-infiltrating NKG2D-CAR-NK92 cells. **G** The expression of IFNγ in tumor-infiltrating NKG2D-CAR-NK92 cells. Data are represented as the mean ± standard error of the mean (SEM). *ns* no significance, *P < 0.05, **P < 0.01 by an unpaired Student’s t-test

We evaluated the ability of two types of NKG2D-CAR-NK cells against PANC28 pancreatic tumor cells in a xenograft mouse model. NSG mice were engrafted s.c. with 5 × 10^6^ tumor cells. Once the tumor volumes had reached approximately 80–100 mm^3^ size, PBS, non-transduced NK92 cells, NC-NKG2D-CAR-NK92 and SH-NKG2D-CAR-NK92 cells (1 × 10^7^ cells/mouse) were injected i.v. into the tumor-bearing mice and each mouse was intraperitoneally injected with 20000 IU of IL-2. The experiment was ended on the 28th day of treatment. SH-NKG2D-CAR-NK92 cells produced remarkable antitumor ability compared with NK92 cells and NC-NKG2D-CAR-NK92 cells in vivo, the tumor volume and weight were lower in the group treated with SH-NKG2D-CAR-NK92 cells than that with NC-NKG2D-CAR-NK92 cells (Fig. 6A–C). Moreover, the statistical results of mouse body weight showed that there was no difference in body weight between groups, indicating the safety of NKG2D-CAR-NK92 cells (Fig. 6D). Furthermore, we detected the proportion of NK cells and the expression of GzmB and IFNγ in tumors and blood. The results revealed a higher proportion of NK cells and higher level of expression of GzmB and IFNγ in NK92 cells from tumors of the mice treated with SH-NKG2D-CAR-NK92 cells (Fig. 6E–G). The similar results were also detected in blood from the mice treated with SH-NKG2D-CAR-NK92 cells (Additional file 1: Fig. S10A–C). These data strongly suggested that *GPR116* downregulation in NKG2D-CAR-NK92 cells could effectively enhance the antitumor efficiency in vivo.

## Discussion

For decades, the development of cancer has been focused on the gene and mutation of tumor cells. However, the progress of cancer is also regulated by tumor microenvironment (TME). TME may provide an important factor to promote cancer development or escape host immune surveillance. Many studies have confirmed the significance of TME immune cells in the development of tumors, and regard it as the target of immunotherapy. Therefore, cytotoxic lymphocytes such as NK and CD8^+^ T cells are important prerequisites for immunotherapy [30]. Natural killer cell is a powerful member of innate lymphocyte family, which has the characteristics of “adaptability” or “training immunity” [31]. During cell transformation or viral infection, they can rapidly respond without antigen specificity. NK cells, as innate lymphoid cells, eliminate tumor cells and release inflammatory cytokines by direct targeting. In recent studies, NK cells, modified NK cells, NK cells combined with T cells or drugs are usually used to treat tumors. For example, the combination of NK cells and tumor reactive T cells or NY-ESO-1 specific TCR-T cells can more effectively inhibit tumor growth [31]. Raja et al. showed that PP4 inhibition via STAT1 and NF- κB signal pathway promotes NK cell-mediated anti-ovarian cancer function [32]. How to enhance immune cell functions in tumors remains a major challenge in cancer immunotherapy.

Studies on several knockout (KO) mouse models found that *GPR116* restricted the infiltration of pulmonary surfactant and alveolar macrophages [33]. In addition, the specific *GPR116* KO model of vascular endothelium shows the leaking blood-brain barrier, suggesting that *GPR116* plays an important role in maintaining the integrity of endothelial cell connection and the formation of subretinal cells [34]. Lu et al. have reported that *GPR116* plays an important role in kidney disease physiology and plays a key role in regulating urine pH value [35]. Although we are more and more aware of the biological significance of *GPR116* receptor in lung and other tissues, its regulation function on immune cells has not been studied [36, 37]. Our study found that both the number of NK cells infiltrating into the tumor and the function of NK cells in tumor were increased in *GPR116* receptor-deficient mice.

NK cells express a large number of variable activation receptors to recognize viral and/or stress-induced cellular ligands, including NKp46, NKp30 and NKG2D [38]. The important inhibitory NK cell receptors are the C-type lectin like heterodimer receptor NKG2A and its subtypes NKG2B and KLRG1 [39]. We found that the expression of *GPR116* receptor was downregulated in activated NK cells. In addition, *GPR116* receptor deletion in NK cells promoted the expression of NKG2D and NKp46, while inhibited the expression of NKG2A and KLRG1. The results showed that there was a negative correlation between *GPR116* receptor and NK cell activation.

The NK cells are activated when encounter target cells and the particles containing perforin are released to form a hole on the membrane of the target cell. Granzymes enter the cytoplasm of tumor target cells through perforin pores. According to the different tumor target cells, the granzymes can induce apoptosis, necrosis or focal death of tumor cells [20, 40–44]. Downregulating of the expression of *GPR116* receptor in NK92 cells promoted the cytotoxicity of NK92 cells by increasing GzmB, IFNγ and CD107a expression. The similar results were discovered in murine *GPR116*^−/−^ NK cells in vitro and in vivo.

NF-κB is not only responsible for regulating inflammation-related genes, but also closely related to diseases such as cancer. He et al. found that human serum albumin-encapsulated black phophorus quantum dots can interact with Toll-like receptors (TLRs) on the surface of NK cells and increase the expression level of mTOR, thus activating the downstream NF-κB signal pathway to regulate cytokine secretion and enhance antitumor function [45]. Wang et al. have reported that both canonical and non-canonical NF-κB pathways are required for NK cell-mediated killing effect [46]. Hypoxia is a common feature of solid tumors. Cells adapt to hypoxia and environment by up-regulating transcription factor HIF1α. Cell RNA sequencing showed that the conditional deletion of HIF1α in tumor-infiltrating NK cells inhibited tumor growth and enriched the expression of NF-κB pathway [47–50]. It is well known that GPCR plays a related role through intracellular G protein, and it has reported that *GPR116* receptor regulates RhoA and Rac1 to affect breast cancer metastasis through Gαq [6]. Our results indicated that GPR116 receptor promoted HIF1α to inhibit the phosphorylation of P65 through Gαq. As we expected, the expression of CD107a, GzmB and IFNγ were suppressed after adding the inhibitor of NF-κB signaling pathway in NK92 cells. The results showed that *GPR116* receptor affected the function of NK cells through Gαq/HIF1α/NF-κB signaling pathway.

Chimeric antigen receptor T cell (CAR-T) therapy has recently showed remarkable efficiency in the treatment of blood tumors [51]. As one of the most effective methods in tumor immunotherapy, it has attracted the attention of many researchers. However, this application had side effects and limitations, such as the risk of graft-versus-host disease (GVHD), cytokine release syndrome (CRS), etc. [52]. NK cell therapy doesn’t have heterologous reaction, making it a ready-made product. CAR-NK cell therapy can overcome some serious limitations of CAR-T cells therapy [53]. In current clinical studies, CXCR4-modified CAR-NK promotes its migration to bone marrow and enhances its anti-multiple osteosarcoma function [54]. At the same time, Du et al. found that NK cells co-expressing IL-15 and NKG2D-CAR can effectively control recurrent or refractory acute myeloid leukemia [55]. In the preclinical study, it was found that CD276-CAR-NK-92 cells showed significantly enhanced cytotoxicity to U-937 or U-937 CD19/tag AML cell lines after a gene knockout of three different inhibitory checkpoints (CBLB, NKG2A, TIGIT) in CAR-NK cells with CRISPR-Cas9 technology [56]. Our previous studies have proved that *GPR116* receptor played a negative role in NK cell function, therefore we modified NKG2D-CAR-NK-92 cells with *GPR116* receptor knockdown to enhance the antitumor activity. The data showed that NKG2D-CAR-NK92 cells with *GPR116* receptor knockdown released more cytotoxic substances such as GzmB and IFNγ, and suppressed tumor growth more effectively both in vitro and in vivo compared to NKG2D-CAR-NK92 cells.

## Conclusions

**Fig. 7:**
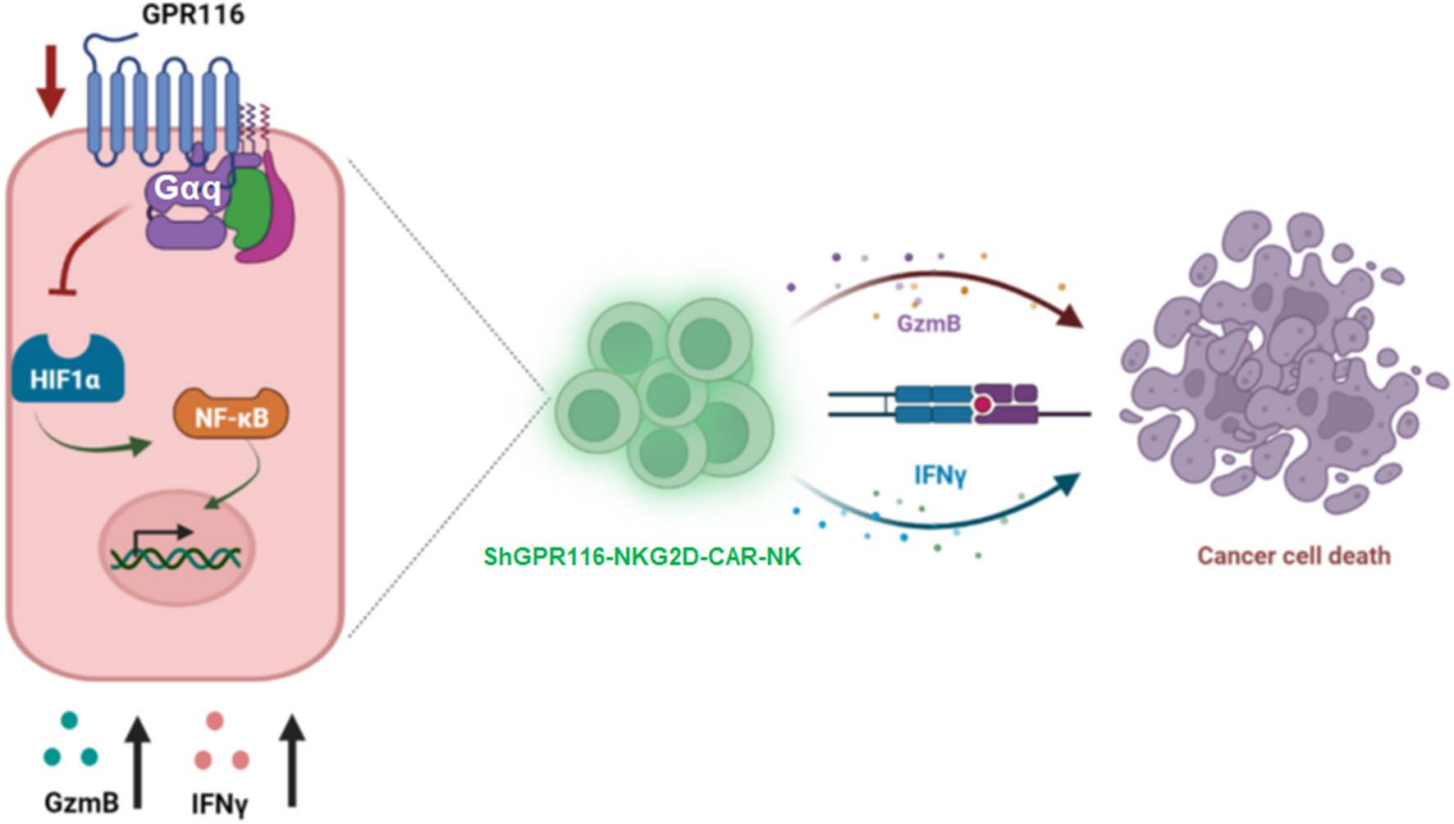
Schematic diagram showing the mechanism by which GRP116 regulates the antitumor function of CAR-NK cell therapy via Gαq/HIF1α/NF-κB signaling pathway

In summary, our results demonstrated that *GPR116* receptor negatively regulated the activation and function of NK cells through Gαq/HIF1α/NF-κB signaling pathway, and knockdown of the expression of *GPR116* receptor could activate NKG2D-CAR-NK92 cells by increasing the release of INF-γ and GzmB and effectively enhance their anti-pancreatic cancer effects (Fig. 7). *GPR116* receptor may be a potential immune checkpoint for NK cells and our study provided a new idea for antitumor therapy with modified CAR-NK cells.

## Methods

### Cells

Human natural killer cell line NK92 was purchased from the American Type Culture Collection and grown in α-MEM medium (Cat. No.12571089; Life Technologies, Carlsbad, CA, USA) supplemented with 12.5% fetal bovine serum (FBS; Cat. No.1614007; Gibco, Grand Island, NY, USA), 12.5% horse serum (Cat. No. 26050070; Gibco), 1.5 g/L sodium bicarbonate, 2 mM L-glutamine (Cat. No. 25030149; Gibco), 100 to 200 U/ml recombinant IL-2 (Cat. No. 200-02; PeproTech, Rocky Hill, NJ, USA,), 0.1 mM 2-mercaptoethanol, 0.2 mM inositol (Cat. No. I5125; Sigma-Aldrich, St. Louis, MO, USA), 0.02 mM folic acid, and 1% penicillin-streptomycin solution (Cat. No. SV30010; Solarbio, Beijing, China). Mouse pancreatic carcinoma cell line PAN02 and human pancreatic carcinoma cell line PANC28, PANC1, SW1990 were purchased from the American Type Culture Collection and cultured in DMEM medium contained 10% FBS (Gibco, Gaithersburg, MD, USA) at 37 °C in an atmosphere of 5% CO_2_.

### Mouse NK cell isolation

Mouse NK cells were isolated from fresh spleens of wild-type (WT) or *GPR116*^−/−^ mice on C57BL/6 background. NK cells were purified with MagniSort Mouse NK cell Enrichment Kit (Invitrogen), followed by CD49b MACS (Miltenyi). The cell population was highly selected for NK cells with a purity of > 99%.

### Quantitative real-time polymerase chain reaction (qRT-PCR)

The NK92 cells, mouse NK cells from the spleens of WT or *GPR116*^−/−^ mice were stimulated by IL-15. After 24 h, the total RNAs were collected using Trizol reagent (Takara, Japan), and then reversely transcribed through the Prime Script RT-PCR kit (Takara, Japan) according to the manufacturer’s instructions. Quantitative real-time PCR was conducted with SYBR Premix Ex Taq (Takara, Japan) on a 7500 Real-time PCR system (Applied Biosystems, Inc., USA).

### Flow cytometry

1 × 10^6^ single suspension cells were stained with the proper antibodies diluted in cell staining buffer (420201, BioLegend, USA) for 30 min at 4 °C. For surface staining, anti-mouse CD45-Alexa Fluor^®^ 700 (Biolegend, 109821), anti-mouse CD11b-PercpCy5.5 (Biolegend, 101227), anti-mouse CD3-PE (Biolegend, 100206), anti-mouse CD3-FITC (Biolegend, 100203), anti-mouse CD4-APC (Biolegend, 100412), anti-mouse CD8-PercpCy5.5 (Biolegend, 100721) and anti-mouse NK1.1-APC (eBioscience, 17-55941-82) were used for extracellular staining. For intracellular staining, the cells were stimulated with or without various cytokines and GolgiPlug for 4 h and then fixed/permeabilized with BD Cytofix/Cytoperm kit as the recommendation of the manufacturer. After washing with wash buffer, anti-human IFN-γ-Percp-cy5.5 (Invitrogen, 45-7319-42), anti-human and Granzyme B-PE (Invitrogen, 12-8899-41) were used for intracellular staining. All samples were determined using FACSalibur flow cytometer (Becton-Dickinson, FL, NJ, USA), and the percentages of cells within each phase of the cell cycle were analyzed using FACS Express 7 software (De Novo Software).

### Western blot

In detail, cells were collected and lysed in radio immunoprecipitation assay (RIPA) buffer (50 mM Tris pH 7.4, 150 mM NaCl, 1 mM EDTA, 1% Nonidet P-40, 0.5% sodium deoxycholine, and 1 mM NaF), and the protein concentration was measured using a BCA protein assay kit (Pierce Biotechnology). Proteins were separated on 10% SDS-polyacrylamide gels and transferred onto polyvinylidene difluoride (PVDF) membranes (Millipore, Bedford, MA, USA). After 2 h, the membranes were blocked with 5% skim milk and incubated with the primary antibodies at 4 °C overnight. After that, the membranes were washed with TBST for three times and incubated with HRP-labeled goat anti-rabbit antibody [Cell Signaling Technology (CST), USA] for 0.5 h. Finally, the immunoreactive signals were detected using the ECL detection system (SuperSignal West Femto Maximum Sensitivity Substrate, Thermo Fisher Scientific, IL, USA). The following primary antibodies were applied: rabbit anti-HIF1α (36169S, CST, USA), rabbit anti-P-p65 (3033S, CST, USA), rabbit anti-*GPR116* (ab136262, Abcam, Shanghai, China), rabbit anti-p65 (8242S, CST, USA), rabbit anti-β-actin (4970 L, CST, USA), rabbit anti-GNA11 (ab153951, abcam, UK).

### Mouse models

WT or *GPR116*^−/−^ mice on C57BL/6 background at the age of 6 to 8 weeks were used in this study. PAN02 cells were suspended at 5 × 10^6^/100 µL PBS and subcutaneously injected into the right back flank of mice to establish subcutaneous tumor models. Tumor volumes were measured and calculated as length × width^2^ × 0.5. One week later, according to tumor size, they were divided into three groups and the mice was injected with PBS, WT-NK and *GPR116*^−/−^-NK through tail vein, respectively.

6 to 8-week-old female NSG were used to establish subcutaneous tumor models to analyze the therapeutic effect of CAR-NK cells in this study. For the cell line PANC28-luc subcutaneous xenograft models, 5 × 10^6^ PANC28-luc cells in 100 μL PBS were subcutaneously inoculated into the right flanks of NSG mice (on day 0). When tumor volume reached approximately 80 mm^3^, the mice were randomly divided into four groups: PBS, NK92, NKG2D-CAR-NK92 and SH-NKG2D-CAR-NK92 (n = 4), and received PBS or 5 × 10^6^ irradiated CAR-NK (NKG2D-CAR-NK92 or SH-NKG2D-CAR-NK92) cells intravenously (every 7 days, 3 times in total). All animal studies were carried out under protocols approved by the Institutional Animal Care and Use Committee of East China Normal University.

### Analysis of tumor-infiltrating immune cell subsets

Tumors were excised and digested postmortem using a cocktail of 1 mg/ml collagenase type IV (Sigma-Aldrich) and 0.02 mg/mL DNaseI (Sigma-Aldrich). After digestion at 37 °C for 30 min, cells were passed through a 70 μm filter twice. Cells were then analyzed for various functional parameters including cytokine production by flow cytometry directly ex vivo as previously described [41].

### Statistics

Data were analyzed using Prism v7.0 (GraphPad Software). Survival curves were analyzed using the log-rank test. Statistically significant differences of P < 0.05, P < 0.01, P < 0.001, and P < 0.0001 are noted with *, ** and ***.

## Supplementary Information

**Additional file 1: Fig. S1** GPR116 deficiency increases the proportion of NK cells in different organs. The different organs were collected and the immune cells were analyzed by flow cytometry. A The proportion of NK cells in liver. B The proportion of NK cells in bone marrow (BM). C The proportion of NK cells in spleen. D The proportion of NK cells in lymph nodes (LN). E The proportion of NK cells in lung. All data are from at least three independent experiments. **Fig. S2** GPR116 receptor inhibites the activation of NK cells. A The expression of GPR116 in NK92 cells was analyzed using qRT-PCR.after treating with IL-15. B The expression of GPR116 in IL-15-stimulated mouse NK cells was analyzed by qRT-PCR. C and D The activating receptor NKG2D and NKP46 expression in WT and GPR116-/- NK cells was analyzed by qRT-PCR. E and F The expression of inhibiting receptor NKG2A and KLRG1 in WT and GPR116-/- NK cells was analyzed by qRT-PCR. All data are from at least three independent experiments. Data are represented as the mean ± standard error of the mean (SEM). *P < 0.05, **P < 0.01, ***P < 0.001 by an unpaired Student’s t-test. **Fig. S3** GPR116 deficiency increases the cytotoxicity of WT and GPR116-/- NK cells in vitro. A and E The cytotoxicity of WT and GPR116-/- NK cells against YAC-1 cells at a different effector to target (E: T) ratios for 4 h. B Flow cytometry analysis of the expression of CD107a in NK cells after co-incubating with YAC-1 cells at a 10:1 ratio for 4h. C and F Flow cytometry analysis of the expression of GzmB in NK cells after co-incubating with YAC-1 cells at a 10:1 ratio for 4h. D and G Flow cytometry analysis of the expression of IFNγ in NK cells after co-incubating with YAC-1 cells at a 10:1 ratio for 4h. All data are from at least three independent experiments. Data are represented as the mean ± standard error of the mean (SEM). *P < 0.05, **P < 0.01, ***P < 0.001 by an unpaired Student’s t-test. **Fig. S4** GPR116 affects HIF1α/NF-κB signaling pathway. A Scatter plots were generated using the Tumor Immune Estimation Resource (TIMER2.0) web. B qRT-PCR analysis of HIF1αexpression in IL-15-stimulated NK92 cells. C qRT-PCR analysis of HIF1α expression in IL-15-stimulated mouse NK cells. D qRT-PCR analysis of HIF1α expression in NK92 cells (NC) and GPR116 knockdown NK92 cells (SH). E Dual luciferase reporter system detected the activation of NF-κB after downregulating GPR116. All data are from at least three independent experiments. Data are represented as the mean ± standard error of the mean (SEM). *P < 0.05, **P < 0.01, ***P < 0.001 by an unpaired Student’s t-test. **Fig. S5** GPR116 affected downstream HIF1α and NF-κB pathway via Gaq. A Western blot analysis of p-P65 and HIF1α in NK92 cells with or without GPR116 or Gαq knockdown. B Flow cytometry analysis of the killing ability of NK92 cells. C Flow cytometry analysis of the expression of GzmB (F), IFNγ (G) and CD107a in NK92 cells with or without GPR116 or Gαq knockdown. All data are from at least three independent experiments. Data are represented as the mean ± standard error of the mean (SEM). *P < 0.05 by an unpaired Student’s t-test. **Fig. S6** Generation and characterization of NKG2D-CAR-NK92 cells with GPR116 knockdown. A Flow cytometry analysis of the transduction efficiencies by staining with fluorescently-labelled anti-NKG2D antibodies. B WB analysis of GPR116 protein level in NK92 cells, NC-NKG2D-CAR-NK92 cells, and SH-NKG2D-CAR-NK92 cells. All data are from at least three independent experiments. Data are represented as the mean ± standard error of the mean (SEM). ns, not significant, **P < 0.01 by an unpaired Student’s t-test. **Fig. S7** Flow cytometry analysis of MICA/B expression in tumor cell lines PANC28, PANC1, Capan2, BXPC3, SW1990. All data are from at least three independent experiments. **Fig. S8** Downregulation of GPR116 receptor enhanced the antitumor activity of NKG2D-CAR-NK92 cells in vitro. A and C Flow cytometry analysis of the expression of CD107a in NK92 cells after co-incubating with PANC1 cells at a 5:1 ratio for 4h. B and D Flow cytometry analysis of the expression of GzmB in NK92 cells after co-incubating with PANC1 cells at a 5:1 ratio for 4h. All data are from at least three independent experiments. Data are represented as the mean ± standard error of the mean (SEM). *P < 0.05, **P < 0.01, ***P < 0.001 by an unpaired Student’s t-test. **Fig. S9** Downregulation of GPR116 receptor enhanced the antitumor activity of NKG2D-CAR-NK92 cells in vitro. A Flow cytometry analysis of the expression of CD107a in NK92 cells after co-incubating with SW1990 cells at a 5:1 ratio for 4h. B Flow cytometry analysis of the expression of GzmB in NK92 cells after co-incubating with PANC1 cells at a 5:1 ratio for 4h. All data are from at least three independent experiments. Data are represented as the mean ± standard error of the mean (SEM). ns, not significant, *P < 0.05 by an unpaired Student’s t-test. **Fig. S10** GPR116 deficiency enhanced the proportion and activation of NKG2D-CAR-NK92 cells in blood. A The proportion of NK92 cells in blood. B The expression of GzmB in blood NK92 cells. C The expression of IFNγ in blood NK92 cells. All data are from at least three independent experiments. Data are represented as the mean ± standard error of the mean (SEM). ns, no significance, *P < 0.05, **P < 0.01, ***P < 0.001 by an unpaired Student’s t-test.

## Data Availability

The datasets used and/or analyzed in the current study are available from the corresponding author upon reasonable request.

## Abbreviations

GPCRs: G protein-coupled receptors
aGPCRs: Adhesive GPCRs
WT: Wild-type
RIPA: Radio immunoprecipitation assay
PVDF: Polyvinylidene difluoride (PVDF)
GzmB: Granzyme B
