# Supplementary material for: GPR116 receptor regulates the antitumor function of NK cells via Gαq/HIF1α/NF-κB signaling pathway as a potential immune checkpoint
Source: Cell Biosci. 2023 Mar 9;13:51. doi: 10.1186/s13578-023-01005-7 (PMC9999509; doi:10.1186/s13578-023-01005-7)
Supplement: Supplementary file 1 — Additional file 1: Fig. S1 GPR116 deficiency increases the proportion of NK cells in different organs. The different organs were collected and the immune cells were analyzed by flow cytometry. A The proportion of NK cells in liver. B The proportion of NK cells in bone marrow (BM). C The proportion of NK cells in spleen. D The proportion of NK cells in lymph nodes (LN). E The proportion of NK cells in lung. All data are from at least three independent experiments. Fig. S2 GPR116 receptor inhibites the activation of NK cells. A The expression of GPR116 in NK92 cells was analyzed using qRT-PCR.after treating with IL-15. B The expression of GPR116 in IL-15-stimulated mouse NK cells was analyzed by qRT-PCR. C and D The activating receptor NKG2D and NKP46 expression in WT and GPR116-/- NK cells was analyzed by qRT-PCR. E and F The expression of inhibiting receptor NKG2A and KLRG1 in WT and GPR116-/- NK cells was analyzed by qRT-PCR. All data are from at least three independent experiments. Data are represented as the mean ± standard error of the mean (SEM). *P < 0.05, **P < 0.01, ***P < 0.001 by an unpaired Student’s t-test. Fig. S3 GPR116 deficiency increases the cytotoxicity of WT and GPR116-/- NK cells in vitro. A and E The cytotoxicity of WT and GPR116-/- NK cells against YAC-1 cells at a different effector to target (E: T) ratios for 4 h. B Flow cytometry analysis of the expression of CD107a in NK cells after co-incubating with YAC-1 cells at a 10:1 ratio for 4h. C and F Flow cytometry analysis of the expression of GzmB in NK cells after co-incubating with YAC-1 cells at a 10:1 ratio for 4h. D and G Flow cytometry analysis of the expression of IFNγ in NK cells after co-incubating with YAC-1 cells at a 10:1 ratio for 4h. All data are from at least three independent experiments. Data are represented as the mean ± standard error of the mean (SEM). *P < 0.05, **P < 0.01, ***P < 0.001 by an unpaired Student’s t-test. Fig. S4 GPR116 affects HIF1α/NF-κB signaling pathway. A [file 13578_2023_1005_MOESM1_ESM.docx]

**Additional file Figures**


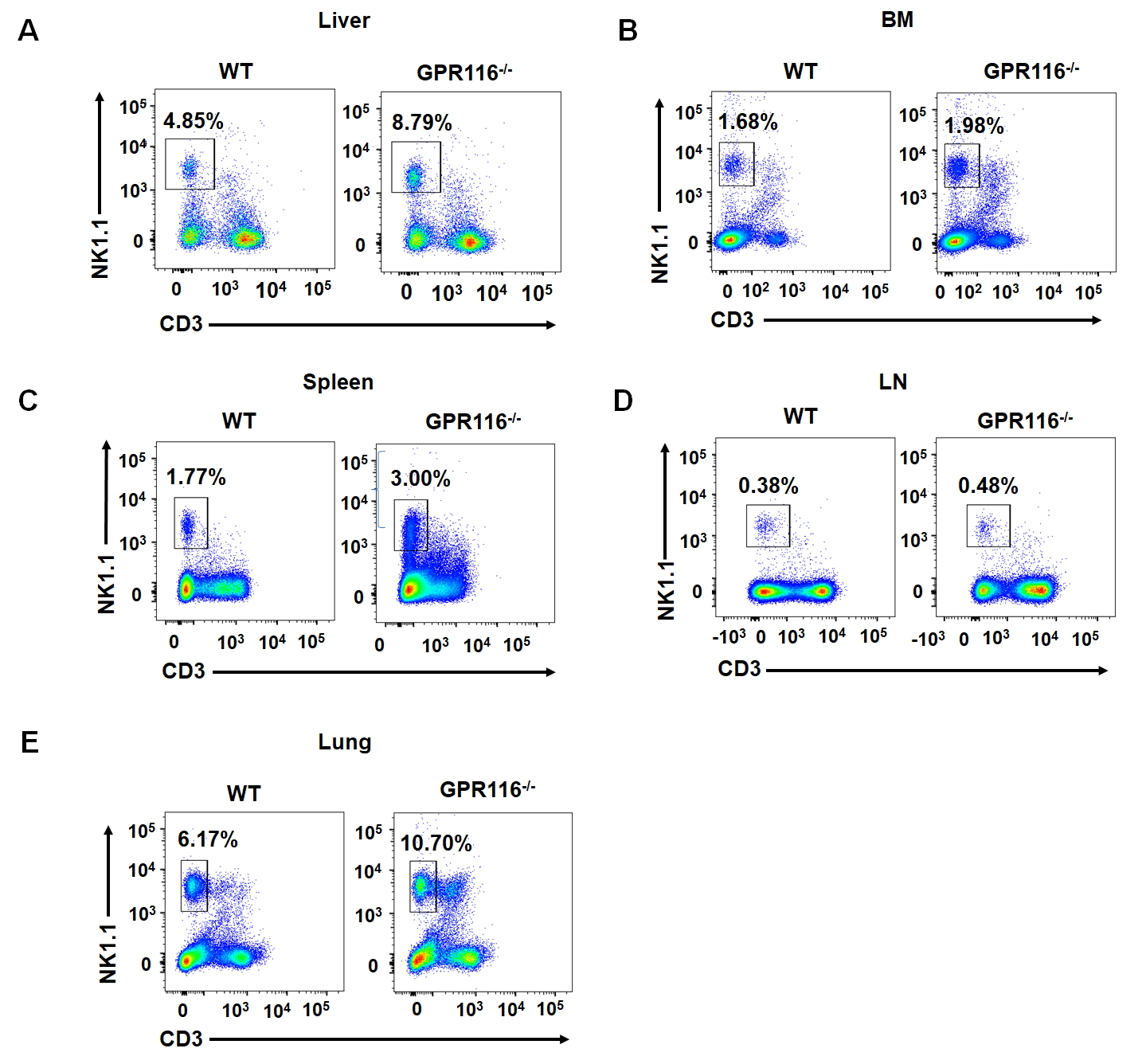


**Fig. S1 GPR116 deficiency increases the proportion of NK cells in different organs.** The different organs were collected and the immune cells were analyzed by flow cytometry. **A** The proportion of NK cells in liver. **B** The proportion of NK cells in bone marrow (BM). **C** The proportion of NK cells in spleen. **D** The proportion of NK cells in lymph nodes (LN). **E** The proportion of NK cells in lung. All data are from at least three independent experiments.


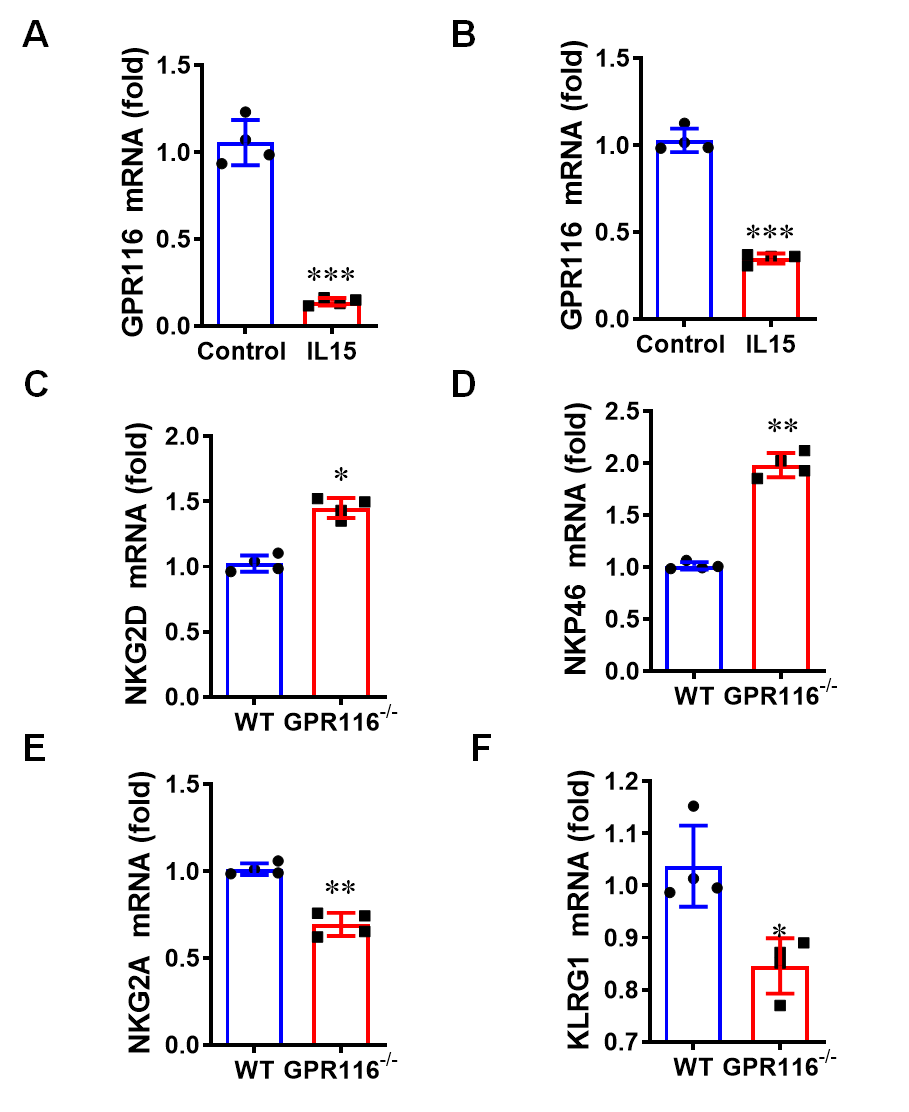


**Fig. S2** **GPR116 receptor inhibites the activation of NK cells.** **A** The expression of GPR116 in NK92 cells was analyzed using qRT-PCR.after treating with IL-15. **B** The expression of GPR116 in IL-15-stimulated mouse NK cells was analyzed by qRT-PCR. **C and D** The activating receptor NKG2D and NKP46 expression in WT and GPR116^-/-^ NK cells was analyzed by qRT-PCR. **E and F** The expression of inhibiting receptor NKG2A and KLRG1 in WT and GPR116^-/-^ NK cells was analyzed by qRT-PCR. All data are from at least three independent experiments. Data are represented as the mean ± standard error of the mean (SEM). *P < 0.05, **P < 0.01, ***P < 0.001 by an unpaired Student’s t-test.


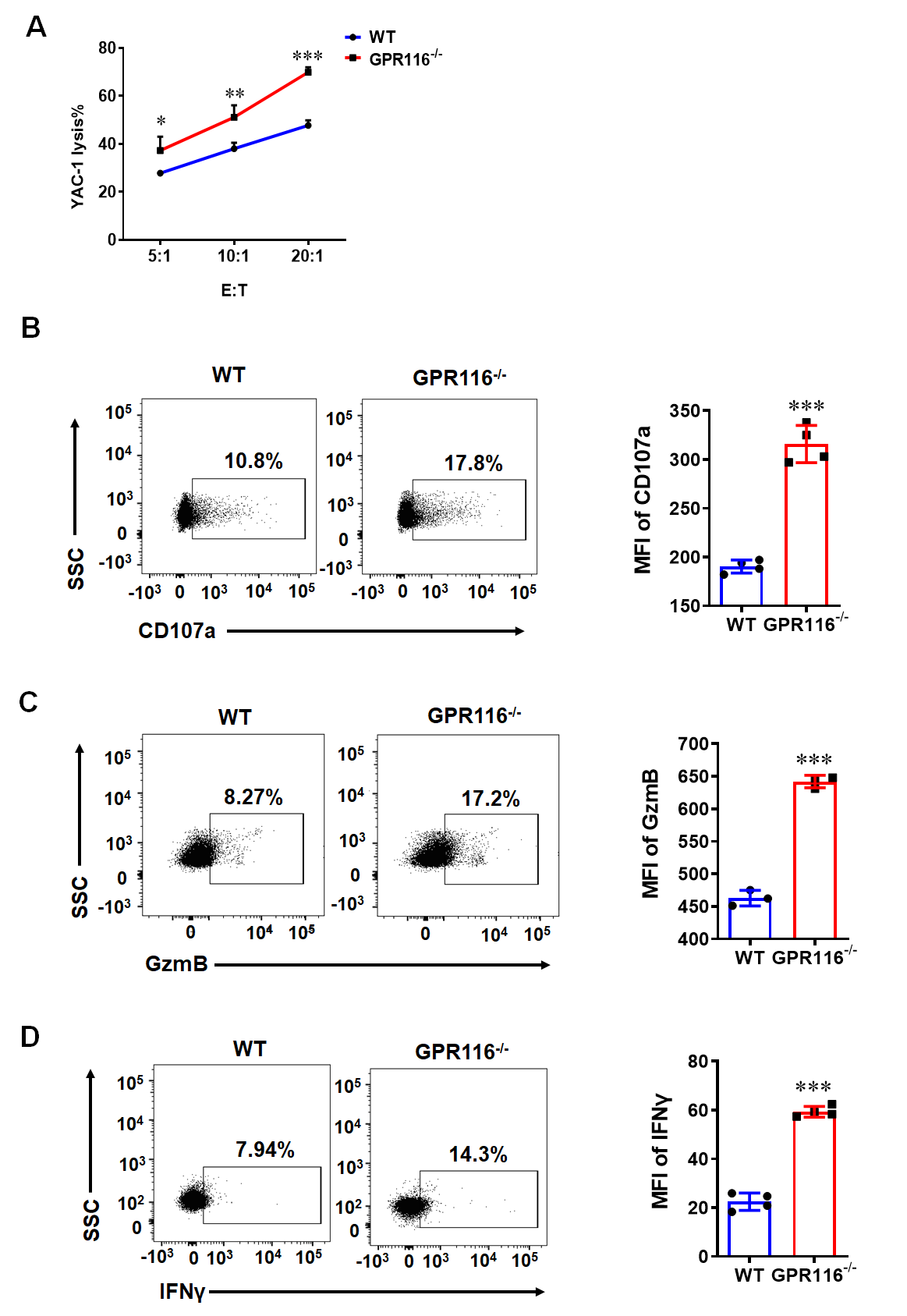


**Fig. S3** **GPR116 deficiency increases the cytotoxicity of WT and GPR116^-/-^ NK cells in vitro.** **A and E** The cytotoxicity of WT and GPR116^-/-^ NK cells against YAC-1 cells at a different effector to target (E: T) ratios for 4 h. **B** Flow cytometry analysis of the expression of CD107a in NK cells after co-incubating with YAC-1 cells at a 10:1 ratio for 4h. **C and F** Flow cytometry analysis of the expression of GzmB in NK cells after co-incubating with YAC-1 cells at a 10:1 ratio for 4h. **D and G** Flow cytometry analysis of the expression of IFNγ in NK cells after co-incubating with YAC-1 cells at a 10:1 ratio for 4h. All data are from at least three independent experiments. Data are represented as the mean ± standard error of the mean (SEM). *P < 0.05, **P < 0.01, ***P < 0.001 by an unpaired Student’s t-test.


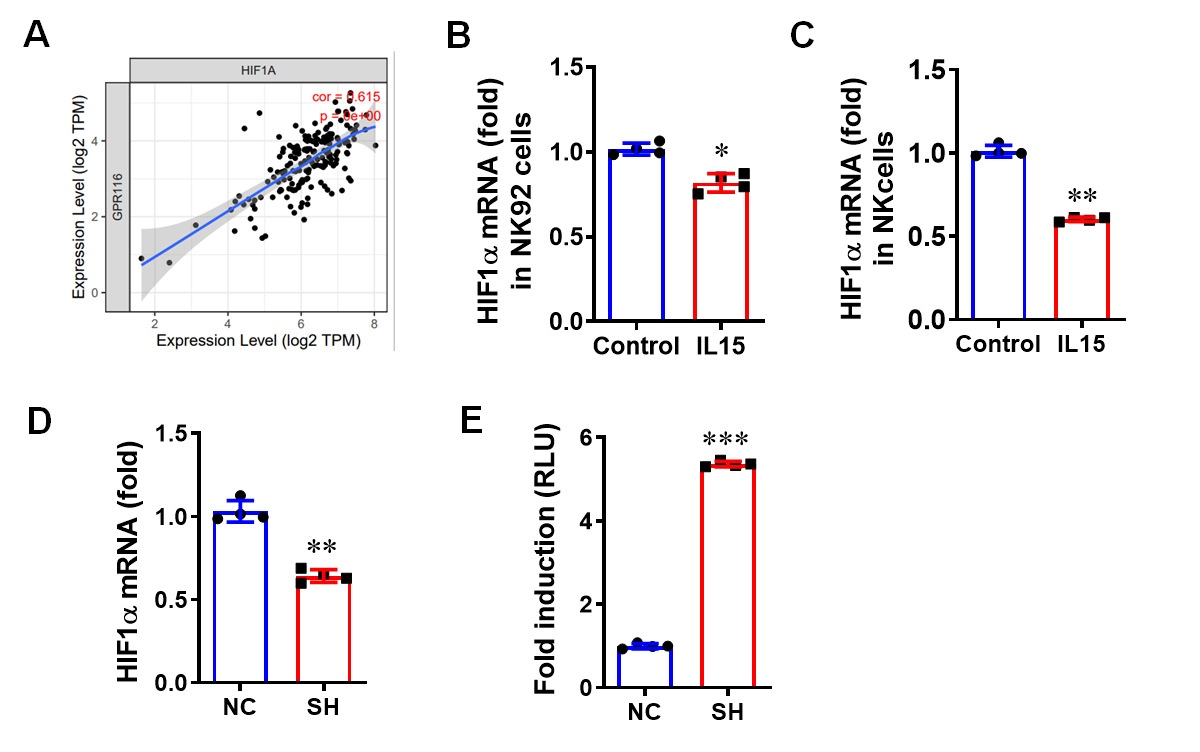


**Fig. S4** **GPR116 affects HIF1α/NF-κB signaling pathway.** **A** Scatter plots were generated using the Tumor Immune Estimation Resource (TIMER2.0) web. **B** qRT-PCR analysis of HIF1αexpression in IL-15-stimulated NK92 cells. **C** qRT-PCR analysis of HIF1α expression in IL-15-stimulated mouse NK cells. **D** qRT-PCR analysis of HIF1α expression in NK92 cells (NC) and GPR116 knockdown NK92 cells (SH). **E** Dual luciferase reporter system detected the activation of NF-κB after downregulating GPR116. All data are from at least three independent experiments. Data are represented as the mean ± standard error of the mean (SEM). *P < 0.05, **P < 0.01, ***P < 0.001 by an unpaired Student’s t-test.


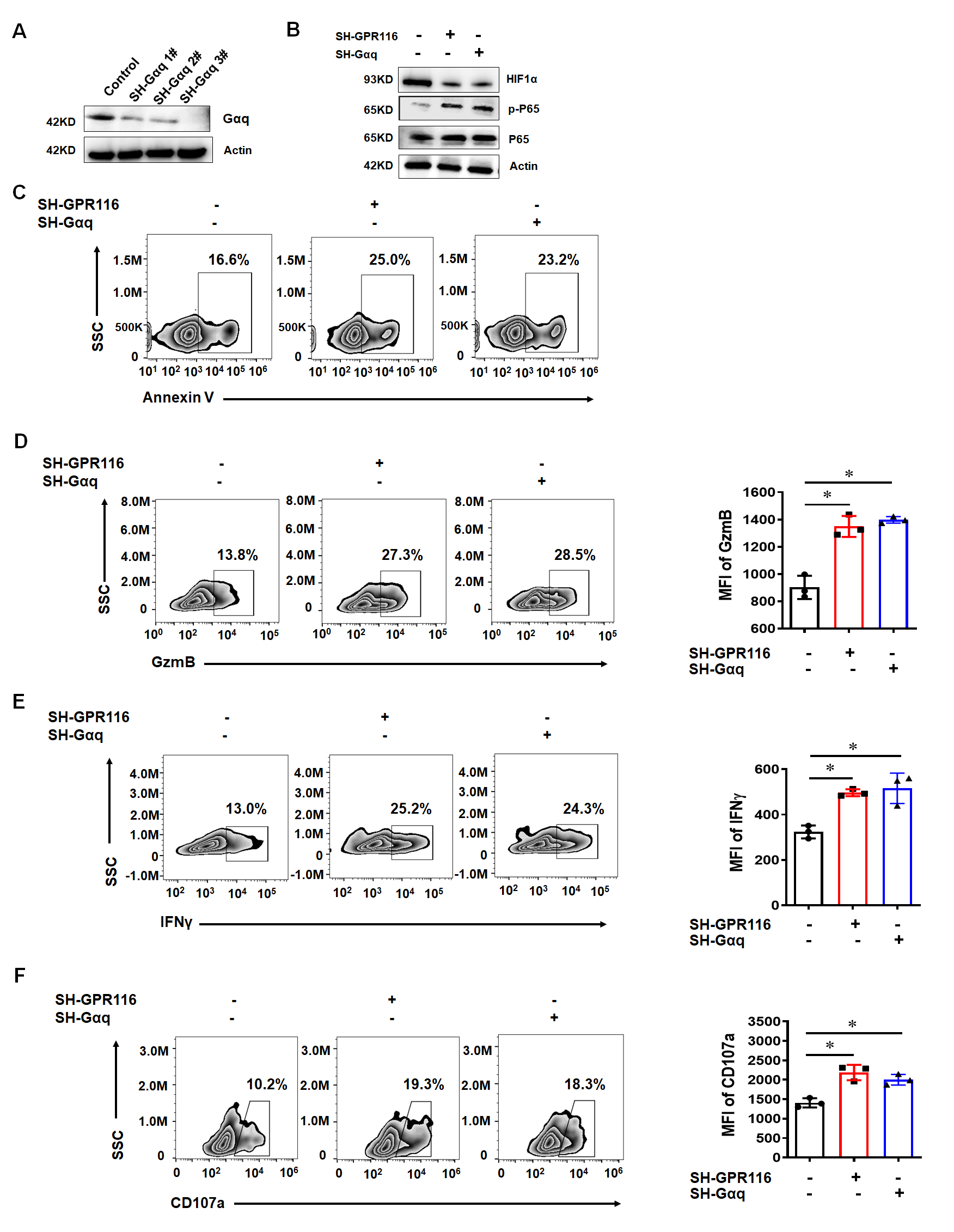


**Fig. S5** **GPR116 affected downstream HIF1α and NF-κB pathway via Gaq.** **A** Western blot analysis of p-P65 and HIF1α in NK92 cells with or without GPR116 or Gαq knockdown. **B** Flow cytometry analysis of the killing ability of NK92 cells. **C** Flow cytometry analysis of the expression of GzmB (F), IFNγ (G) and CD107a in NK92 cells with or without GPR116 or Gαq knockdown. All data are from at least three independent experiments. Data are represented as the mean ± standard error of the mean (SEM). *P < 0.05 by an unpaired Student’s t-test.


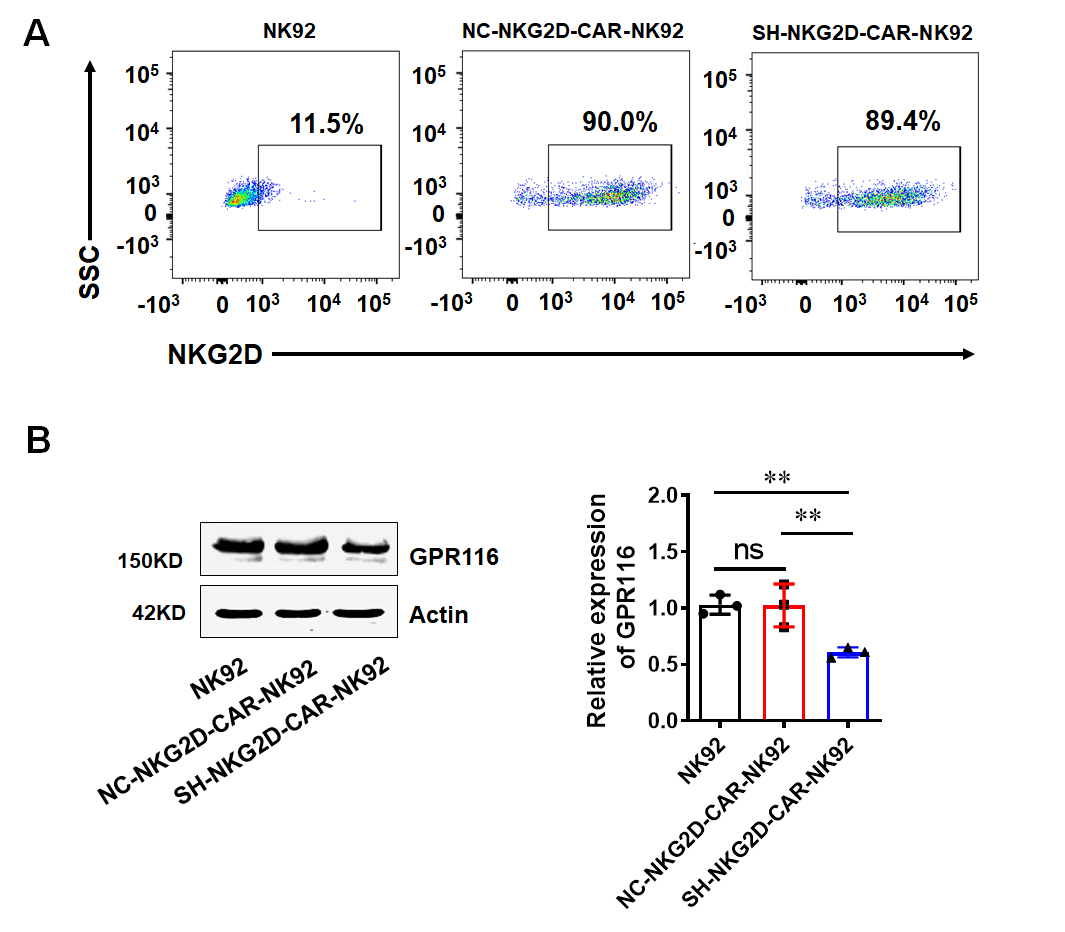


**Fig. S6** G**eneration and characterization of NKG2D-CAR-NK92 cells with GPR116 knockdown.** **A** Flow cytometry analysis of the transduction efficiencies by staining with fluorescently-labelled anti-NKG2D antibodies. **B** WB analysis of GPR116 protein level in NK92 cells, NC-NKG2D-CAR-NK92 cells, and SH-NKG2D-CAR-NK92 cells. All data are from at least three independent experiments. Data are represented as the mean ± standard error of the mean (SEM). ns, not significant, **P < 0.01 by an unpaired Student’s t-test.


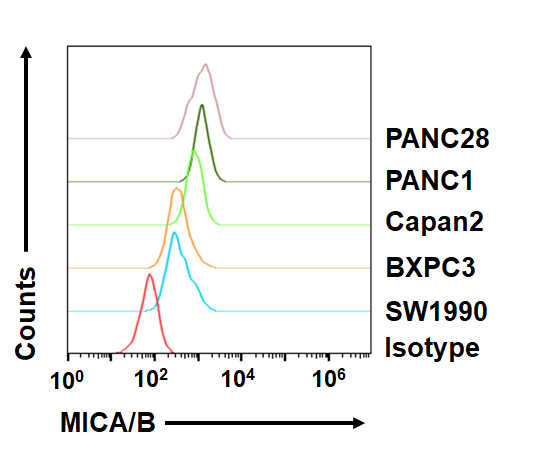


**Fig.** **S7** Flow cytometry analysis of MICA/B expression in tumor cell lines PANC28, PANC1, Capan2, BXPC3, SW1990. All data are from at least three independent experiments.


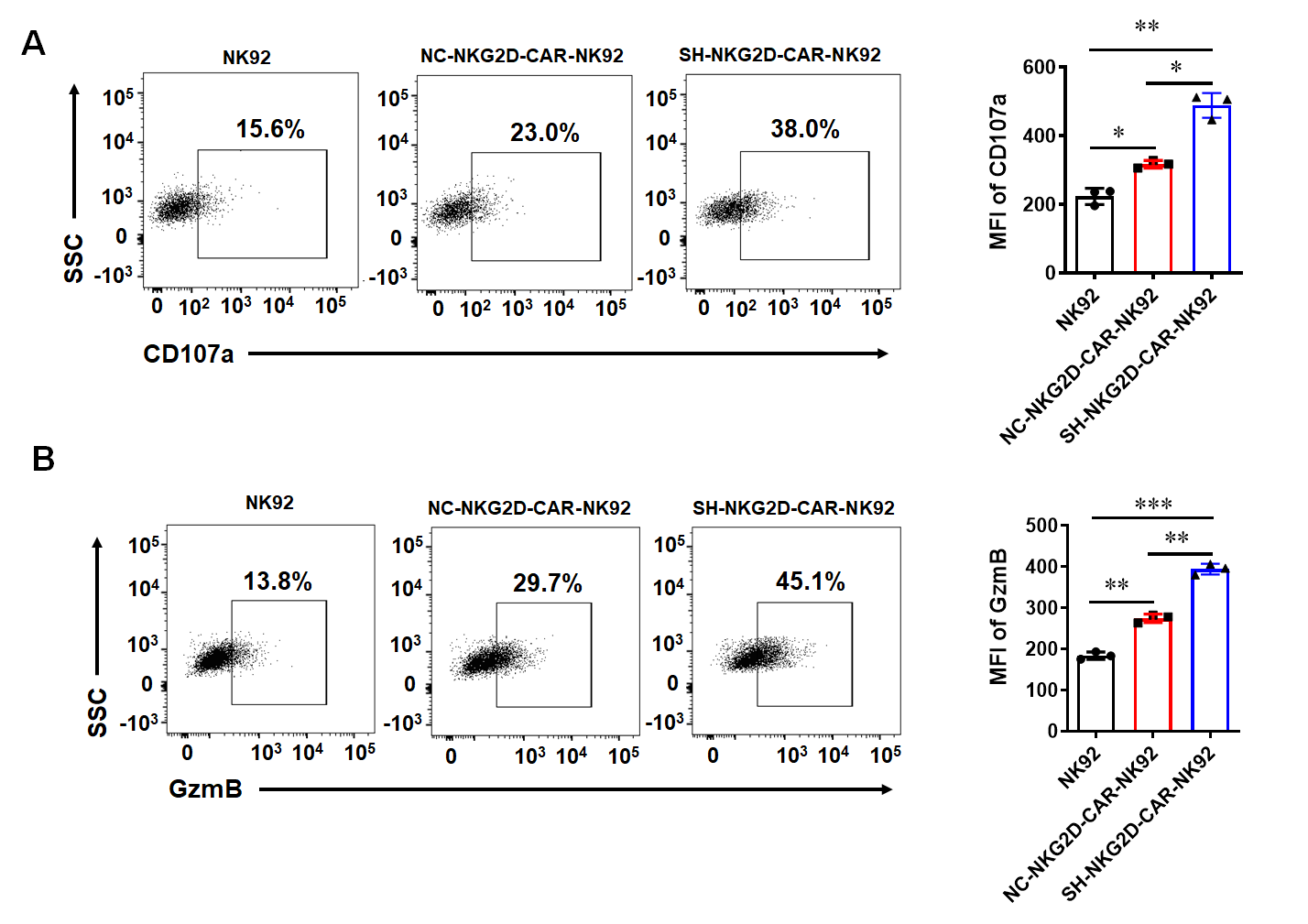


**Fig. S8 Downregulation of GPR116 receptor enhanced the antitumor activity of NKG2D-CAR-NK92 cells in vitro. A and C** Flow cytometry analysis of the expression of CD107a in NK92 cells after co-incubating with PANC1 cells at a 5:1 ratio for 4h. **B** **and D** Flow cytometry analysis of the expression of GzmB in NK92 cells after co-incubating with PANC1 cells at a 5:1 ratio for 4h. All data are from at least three independent experiments. Data are represented as the mean ± standard error of the mean (SEM). *P < 0.05, **P < 0.01, ***P < 0.001 by an unpaired Student’s t-test.


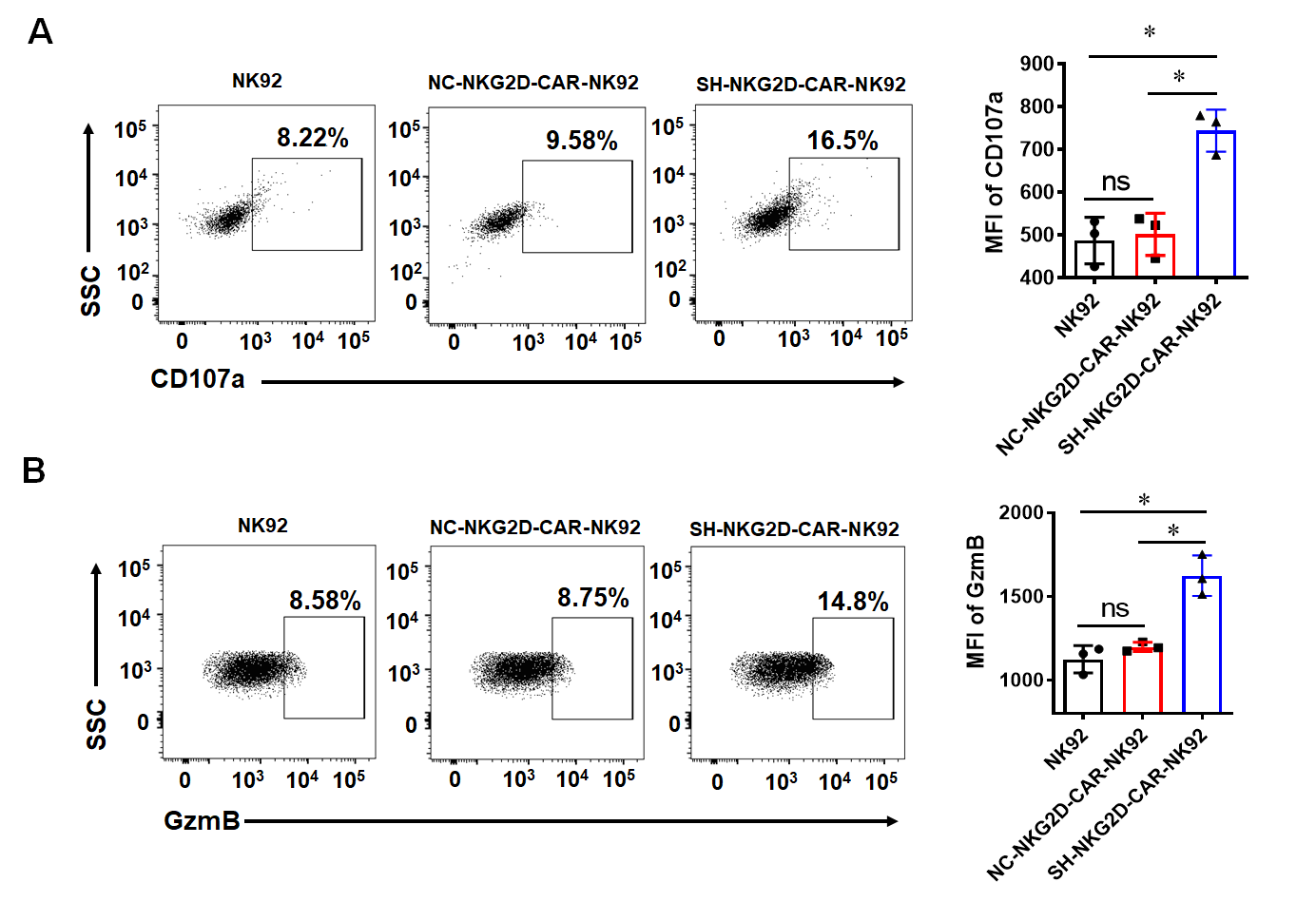


**Fig. S9 Downregulation of GPR116 receptor enhanced the antitumor activity of NKG2D-CAR-NK92 cells in vitro. A** Flow cytometry analysis of the expression of CD107a in NK92 cells after co-incubating with SW1990 cells at a 5:1 ratio for 4h. **B** Flow cytometry analysis of the expression of GzmB in NK92 cells after co-incubating with PANC1 cells at a 5:1 ratio for 4h. All data are from at least three independent experiments. Data are represented as the mean ± standard error of the mean (SEM). ns, not significant, *P < 0.05 by an unpaired Student’s t-test.


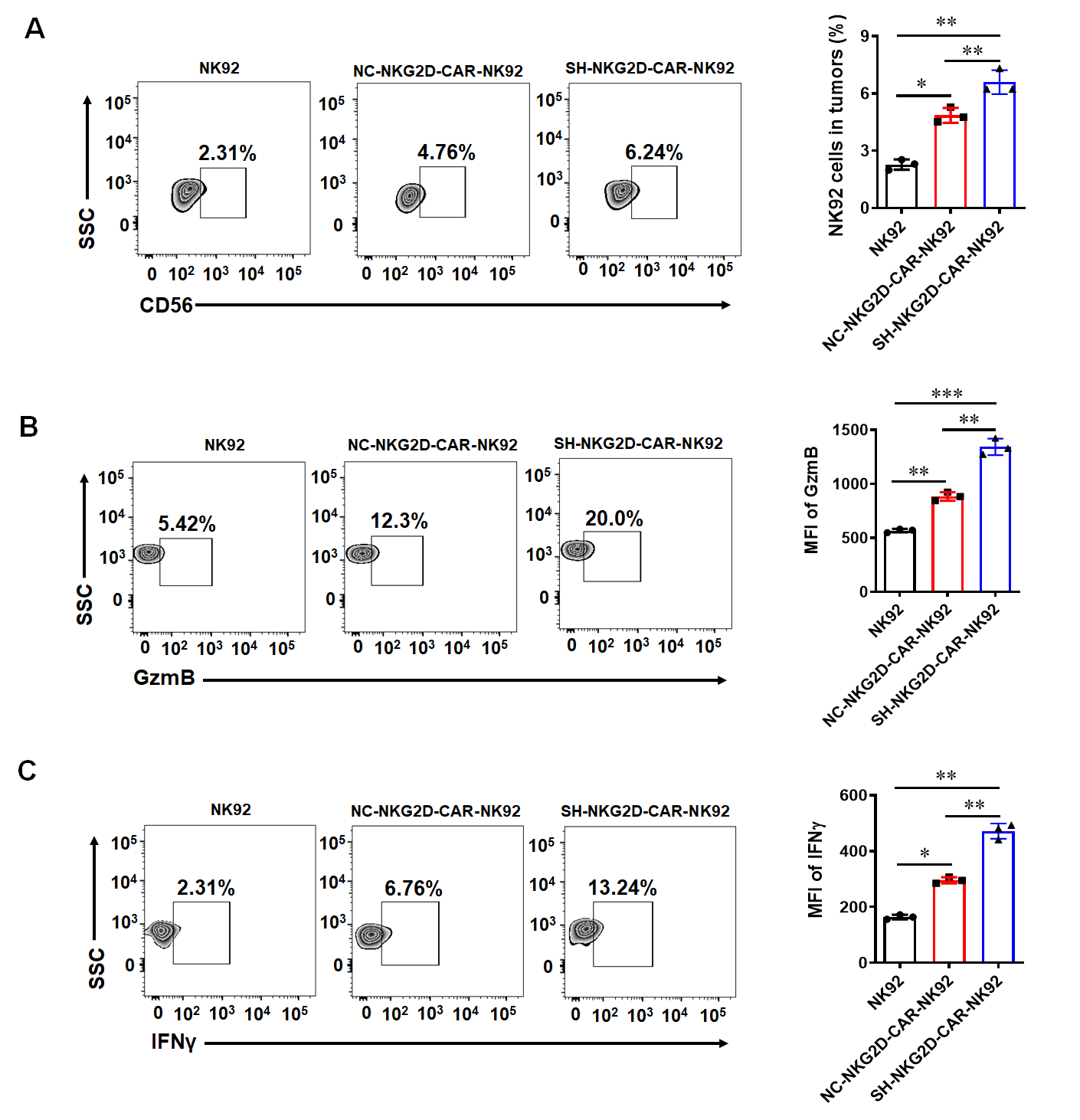


**Fig.** **S10** **GPR116 deficiency enhanced the proportion and activation of NKG2D-CAR-NK92 cells in blood.** **A** The proportion of NK92 cells in blood. **B** The expression of GzmB in blood NK92 cells. **C** The expression of IFNγ in blood NK92 cells. All data are from at least three independent experiments. Data are represented as the mean ± standard error of the mean (SEM). ns, no significance, *P < 0.05, **P < 0.01, ***P < 0.001 by an unpaired Student’s t-test.
